# Associations between three common single nucleotide polymorphisms (rs266729, rs2241766, and rs1501299) of *ADIPOQ* and cardiovascular disease: a meta-analysis

**DOI:** 10.1186/s12944-018-0767-8

**Published:** 2018-05-28

**Authors:** Joseph Sam Kanu, Shuang Qiu, Yi Cheng, Ri Li, Changgui Kou, Yulu Gu, Ye Bai, Jikang Shi, Yong Li, Yunkai Liu, Yaqin Yu, Yawen Liu

**Affiliations:** 1 Department of Epidemiology and Biostatistics, School of Public Health of Jilin University, 1163 Xinmin Street, Changchun, 130021 China; 2 The Cardiovascular Center, the First Hospital of Jilin University, Changchun, 130021 China

**Keywords:** *ADIPOQ*, Single nucleotide polymorphisms, Cardiovascular disease, Association, Meta-analysis

## Abstract

**Background:**

Inconsistencies have existed in research findings on the association between cardiovascular disease (CVD) and single nucleotide polymorphisms (SNPs) of *ADIPOQ*, triggering this up-to-date meta-analysis.

**Methods:**

We searched for relevant studies in PubMed, EMBASE, Cochrane Library, CNKI, CBM, VIP, and WanFang databases up to 1st July 2017. We included 19,106 cases and 31,629 controls from 65 published articles in this meta-analysis. STATA 12.0 software was used for all statistical analyses.

**Results:**

Our results showed that rs266729 polymorphism was associated with the increased risk of CVD in dominant model or in heterozygote model; rs2241766 polymorphism was associated with the increased risk of CVD in the genetic models (allelic, dominant, recessive, heterozygote, and homozygote). In subgroup analysis, significant associations were found in different subgroups with the three SNPs. Meta-regression and subgroup analysis showed that heterogeneity might be explained by other confounding factors. Sensitivity analysis revealed that the results of our meta-analysis were stable and robust. In addition, the results of trial sequential analysis showed that evidences of our results are sufficient to reach concrete conclusions.

**Conclusions:**

In conclusion, our meta-analysis found significant increased CVD risk is associated with rs266729 and rs2241766, but not associated with rs1501299.

**Electronic supplementary material:**

The online version of this article (10.1186/s12944-018-0767-8) contains supplementary material, which is available to authorized users.

## Background

Cardiovascular disease (CVD) is the primary cause of death worldwide, leading to 32% of all deaths worldwide in 2013 [1]. Epidemiological and biological evidences demonstrate that multiple environmental and genetic factors are implicated in CVD, although the etiology of CVD has not been fully elucidated [2–5]. Identifying CVD-relative risk factors is critical in control of the development and progress of CVD.

Adiponectin is involved in CVD: low levels of adiponectin (hypoadipoectinemia) positively correlate with the risk of CVD, and higher levels of adiponectin protect against this disease [6–11]. Adiponectin is synthesized and secreted by adipose tissue [12], osteoblasts [13], skeletal muscle [14], and cardiomyocytes [15]. This protein, as one of the most abundant adipocytokines in blood, has anti-atherogenic, cardioprotective, anti-inflammatory, and antithrombotic properties [16–20].

Adiponectin is encoded by *ADIPOQ* which is located in chromosome 3q27 [21], and adiponectin levels are influenced by single-nucleotide polymorphisms (SNPs) in *ADIPOQ* [22]. SNPs in *ADIPOQ* have been found to be associated with CVD [23, 24], diabetes [25, 26], stroke [27, 28], myocardial infarction [29, 30], cancer [31, 32], kidney disease [33, 34], and even gynecological conditions [35, 36]. Previous studies have shown the association between SNPs in *ADIPOQ* (rs3774261, rs1063537, rs2082940, rs2241766, rs266729, and rs1501299) and CVD/subclinical CVD [30, 37, 38]. The three common SNPs of *ADIPOQ* (rs266729, rs2241766, and rs1501299) were most widely studied. However, findings from previous studies on the three SNPs in relation to CVD risk are inconsistent and inconclusive.

For rs266729 (− 11,377 C/G) in *ADIPOQ*, Du et al. [39] and Zhang et al. [40] found that the SNP is associated with CVD risk; Stenvinkel et al. [41] revealed that rs266729 is associated with the decreased risk of CVD; Zhang et al. [40], Cheong et al. [27], and Chiodini et al. [29] found that there is no significant association between rs266729 and CVD. For rs2241766 (+ 45 T/G), Pischon et al. [42] and Jung et al. [43] identified no association between rs2241766 and the risk of coronary artery disease (CAD) in patients with type 2 diabetic mellitus (T2DM); Du et al. [39], Oliveira et al. [44], and Mofarrah et al. [45] found that there is a significant association between rs2241766 polymorphism and CAD risk; Chang et al. [46] revealed that rs2241766 is associated with the decreased risk of CVD. Moreover, for rs1501299 (+ 276 G / T), Bacci et al. [47] and Esteghamati et al. [48] revealed that rs1501299 is associated with the decreased risk of CAD; Mohammadzadeh et al. [38], however, reported that there is an association between rs1501299 and CAD risk; Foucan et al. [49] found that there is no significant association between rs1501299 and CAD in patients with T2DM. Thus, those results are inconsistent.

Meta-analysis performed by Zhang et al. in 2012 revealed that associations between the SNPs (rs2241766, rs1501299, and rs266729) in *ADIPOQ* and CVD were significant but weak [50]. Since that data, several more studies have emerged to investigate the association between SNPs in *ADIPOQ* and susceptibility to CVD [37, 38, 45]. In this study, we further collected references and updated meta-analysis of association between SNPs (rs2241766, rs1501299, and rs266729) in *ADIPOQ* and CVD in order to get a more precise and reliable assessment of the association.

## Methods

### Search strategy

We performed an extensive literature search in PubMed, EMBASE, Cochrane Library, CNKI, CBM, VIP, and WanFang databases for published articles on the association between *ADIPOQ* polymorphisms and CVD risk up to July 1st, 2017. The literature search was done without any language or population restrictions imposed. During the literature search, we used various combinations of keywords, such as ‘coronary heart disease (CHD)’ or ‘coronary artery disease’ or ‘cardiovascular disease’ or ‘ischemic heart disease’ or ‘angina’ or ‘myocardial infarction (MI)’ or ‘stroke’ or ‘atherosclerosis’ or ‘arteriosclerosis’ or ‘coronary stenosis’ combined with ‘*ADIPOQ*’ or ‘*APM1*’ or ‘*ACDC*’ or ‘adiponectin gene’ and ‘polymorphisms’ or ‘variants’ or ‘variations’. Joseph Sam Kanu and Shuang Qiu independently performed the literature search for potential articles included in this meta-analysis. All articles retrieved were first organized in reference manager software (Endnote 6).

### Inclusion and exclusion criteria

A study included in this meta-analysis was based on the following criteria: 1) the study has sufficient data to allow association between CVD risk and *ADIPOQ* SNP to be assessed; 2) the study included original data (independence among studies); 3) evaluation of the *ADIPOQ* polymorphisms (rs266729, rs2241766, and rs1501299) and CVD risk; 4) the language of the study was English or Chinese; and 5) observed genotype frequencies in controls must be consistent with Hardy–Weinberg equilibrium (HWE). We excluded a study based on: 1) the study contained overlapping data; 2) the study with missing information (particularly genotype distributions), after corresponding author, who was contacted by us with email, failed to provide the required information; and 3) genome scans investigating linkages with no detailed genotype distributions between cases and controls. Where there was a disagreement on the selection of a study, the issue was resolved by discussion or consensus with the third investigator (Ri Li). For articles with missing data, we emailed the corresponding authors for the required data.

### Assessment of study quality

We used the NATURE-published guidelines proposed by the NCI-NHGRI Working Group on Replication in Association Studies for assessing the quality of each study included in this meta-analysis [51]. These guidelines have a checklist of 53 conditions for authors, journal editors, and referees to interpret data and results of genome-wide or other genotype–phenotype association studies clearly and unambiguously. We used the first set of 34 conditions in assessing the quality of each study. We allocated a score of 1 point for each condition a study met, and no point (0 score) if the condition or requirement is lacking. Each study was given a total Quality Score – the sum of all points each study obtained. Study quality assessment was independently carried out by Joseph Sam Kanu and Shuang Qiu.

### Data extraction

Joseph Sam Kanu and Shuang Qiu extracted data from each study independently. We summarized the information extracted from each article in Table 1. The characteristics of articles included first author, year of publication, country in which the study was done, study population (ethnicity), numbers of cases and controls, genotyping method, SNPs investigated, genotype frequency of cases and controls, and outcome (Table 1; Additional file 1: Tables S1, S2, and S3).

**Table 1:** Characteristics of included studies

Study	ID	Year	Country	Population	Outcome	Sample size		Genotyping Method	Quality Score
						Cases	Controls		
Lacquemant Swiss^70^	1	2004	Switzerland	European	CAD	107	181	Other	9
Lacquemant French^70^	2	2004	France	European	CAD	55	134	Other	9
Bacci^47^	3	2004	Italy	European	CAD	142	234	Other	8
Ohashi^71^	4	2004	Japan	East Asian	CAD	383	368	TaqMan	7
Stenvinkel^41^	5	2004	America	European	CVD	63	141	Other	6
Filippi^72^	6	2005	Italy	European	CAD	580	466	Other	9
Ru Y^73^	7	2005	China	East Asian	CHD	131	136	TaqMan	6
Qi1^74^	8	2005	America	European	CVD	239	640	TaqMan	10
Qi2^24^	9	2006	America	European	CVD	285	704	TaqMan	10
Wang JN^75^	10	2006	China	East Asian	CHD	120	131	PCR-RFLP	7
Hegener 1^76^	11	2006	America	European	MI	341	341	TaqMan	11
Hegener 2^76^	12	2006	America	European	Stroke	259	259	TaqMan	11
Jung^43^	13	2006	Korea	East Asian	CAD	88	68	TaqMan	8
Gable 1^77^	14	2007	UK	European	CVD	266	2,727	PCR-RFLP	11
Gable 2^77^	15	2007	UK	European	MI	530	564	PCR-RFLP	12
Pischon^42^	16	2007	America	European	CHD	1,036	2,071	TaqMan	11
Lu F^78^	17	2007	China	East Asian	CHD	135	131	PCR-RFLP	7
Hoefle^79^	18	2007	Austria	European	CHD	277	125	TaqMan	7
Yamada^80^	19	2008	Japan	East Asian	ACI	313	971	Other	9
Oguri^81^	20	2009	Japan	East Asian	MI	773	1,114	Other	10
Chang^46^	21	2009	China	East Asian	CAD	600	718	PCR-RFLP	9
Zhang XL^82^	22	2009	China	East Asian	CHD	205	135	PCR-RFLP	8
Zhong C^83^	23	2010	China	East Asian	CAD	198	237	TaqMan	10
Foucan 1^84^	24	2010	France	African	CAD	57	159	TaqMan	7
Xu L^85^	25	2010	China	East Asian	CHD	153	73	PCR-RFLP	8
Chiodini^29^	26	2010	Italy	European	MI	503	503	TaqMan	10
Persson^86^	27	2010	Sweden	European	MI	244	244	TaqMan	9
Chen XL^87^	28	2010	China	East Asian	Stroke	357	345	TaqMan	8
Luo SX^88^	29	2010	China	East Asian	CHD	221	100	PCR-RFLP	8
Caterina^89^	30	2011	Italy	European	MI	1,864	1,864	Other	13
Al-Daghri^90^	31	2011	Saudi A.	West Asian	CAD	123	295	PCR-RFLP	8
Prior^91^	32	2011	UK	European	CHD	85	298	PCR-RFLP	7
Leu^92^	33	2011	China	East Asian	Stroke	80	3,330	Other	10
Liu F^28^	34	2011	China	East Asian	Stroke	302	338	PCR-RFLP	9
Rodriguez^93^	35	2011	Spain	European	CVD	119	555	TaqMan	9
Chen F^94^	36	2011	China	East Asian	CHD	93	102	PCR-RFLP	8
Maimaitiyiming^95^	37	2011	China	East Asian	CHD	196	124	PCR-RFLP	8
Hu HH^96^	38	2011	China	East Asian	CHD	150	152	Other	8
Zhang YM^97^	39	2011	China	East Asian	CHD	149	167	PCR-RFLP	8
Zhou NN^98^	40	2011	China	East Asian	CAD	358	65	PCR-RFLP	8
Sabouri^99^	41	2011	UK	European	CAD	329	106	PCR-RFLP	8
Boumaiza^100^	42	2011	Tunisia	African	CAD	212	104	PCR-RFLP	10
Chengang^101^	43	2012	China	East Asian	CAD	267	250	PCR-RFLP	8
Esteghamati^48^	44	2012	Iran	West Asia	CAD	114	127	PCR-RFLP	10
Gui^102^	45	2012	China	East Asian	CAD	438	443	TaqMan	10
Katakami^23^	46	2012	Japan	East Asian	CVD	213	2,424	Other	12
Oliveira^44^	47	2012	Brazil	European	CAD	450	153	Other	10
Shi KL^103^	48	2012	China	East Asian	CAD	396	292	Other	8
Zhang HF^104^	49	2012	China	East Asian	ATHERO	394	118	PCR-RFLP	8
Nannan^105^	50	2012	China	East Asian	CAD	213	467	Other	10
Antonopoulos^106^	51	2013	Greece	European	CAD/MI	462	132	Other	11
Rizk^107^	52	2013	Qatar	West Asian	ACS/MI	142	122	Other	12
Wang CH^108^	53	2013	China	East Asian	CAD	101	116	TaqMan	9
Wu/276^109^	54	2013	China	East Asian	CHD	188	200	PCR-RFLP	9
Cheung^110^	55	2014	China	East Asian	CHD	184	2,012	Other	11
Foucan 2^49^	56	2014	France	African	CAD	54	146	TaqMan	8
Shaker^30^	57	2014	Egypt	African	MI	60	60	PCR-RFLP	8
Li Yang^111^	58	2014	China	East Asian	CAD	234	365	PCR-RFLP	8
Alehagen^112^	59	2015	Sweden	European	ATHERO	105	371	TaqMan	6
Torres^113^	60	2015	Portugal	European	ATHERO	43	263	Other	7
Zhang M^114^	61	2015	China	East Asian	CAD	563	412	Other	11
Liu Yun^115^	62	2015	China	East Asian	CAD	200	200	PCR-RFLP	7
Du SX^39^	63	2016	China	East Asian	CAD	493	304	PCR-RFLP	9
Mofarrah^45^	64	2016	Iran	West Asia	CAD	152	72	Other	8
Mohammadzadeh^38^	65	2016	Iran	West Asia	CAD	100	100	PCR-RFLP	9
Suo SZ^116^	66	2016	China	East Asian	CAD	128	130	PCR-RFLP	9
Zhang Min^40^	67	2016	China	East Asian	MI	306	412	Other	9
Li SS^117^	68	2017	China	East Asian	Stroke	385	418	PCR-RFLP	10

*ACI* atherothrombotic cerebral infarction, *ACS* Acute Coronary Syndrome, *ATHERO* Atherosclerosis, *CAD* coronary artery disease, *CHD* coronary heart disease, *CVD* cardiovascular disease, *IHD* ischemic heart disease, *MI* myocardial infarction

The 70-117 references are listed in Additional file 4

### Statistical analysis

HWE was evaluated for each study using Goodness of fit Chi-square test in control groups, and *P* < 0.05 was considered as a significant deviation from HWE. The strength of association between the three *ADIPOQ* polymorphisms and CVD susceptibility was assessed using odds ratios (*OR*) and 95% confidence intervals (95% *CI*). The associations were measured based on five different genetic models: allelic model (rs266729: G versus C; rs2241766: G versus T; rs1501299: T versus G), dominant model (rs266729: GG + GC versus CC; rs2241766: GG + GT versus TT; rs1501299: TT + TG versus GG), recessive model (rs266729: GG versus GC + CC; rs2241766: GG versus GT + TT; rs1501299: TT versus TG + GG), heterozygote model (rs266729: GC versus CC; rs2241766: GT versus TT; rs1501299: TG versus GG), and homozygote model (rs266729: GG versus CC; rs2241766: GG versus TT; rs1501299: TT versus GG). Heterogeneity were evaluated by the Chi-square test based Q-statistic, and quantified by *I*^*2*^-statistic [52]. If there was no substantial statistical heterogeneity (*P* > 0.10, *I*^*2*^ ≤ 50%), data were pooled by fixed-effect model (Mantel and Haenszel methods); otherwise, the heterogeneity was evaluated by random-effect model (DerSimonian and Laird methods). Meta-regression analysis was performed to detect main sources of heterogeneity. In addition, subgroup analyses were stratified by population (European, East Asian, West Asian, and African), genotyping method (PCR-RFLP, TaqMan, and Others), sample size (< 1000 and ≥ 1000), and quality score (< 10 and ≥ 10). Sensitivity analysis was performed to examine stability of our results by omitting each study in each turn. Publication bias was measured by funnel plots [53], and quantified by the Begg’s and Egger’s tests [54] (*P* < 0.05 considered statistically significant publication bias). STATA 12.0 software (StataCorp. 2011. Stata Statistical Software: Release 12. College Station, TX: StataCorp LP) was used for all statistical analyses. *P*-value < 0.05 was considered statistically significant, except where other-wise specified. A separate analysis was performed for each SNPs included in the meta-analysis.

### Trial sequential analysis (TSA)

Traditional meta-analysis may result in type I and type II errors owing to dispersed data and repeated significance testing [55, 56]. To reduce the risk of type I error, TSA was used to estimate required information size (RIS) and confirm statistical reliability with an adjusted threshold for statistical significance [57]. In present meta-analysis, we used trial sequential analysis software (TSA, version 0.9; Copenhagen Trial Unit, Copenhagen, Denmark, 2011) by setting an overall type I error of 5%, a statistical test power of 80%, and a relative risk reduction of 20% [58, 59].

If the Z-curve crosses trial sequential monitoring boundary or RIS has been reached, a sufficient level of evidence has been reached and further studies are unneeded; otherwise, additional studies are needed to reach a sufficient conclusion.

## Results

### Overall results

**Fig. 1:**
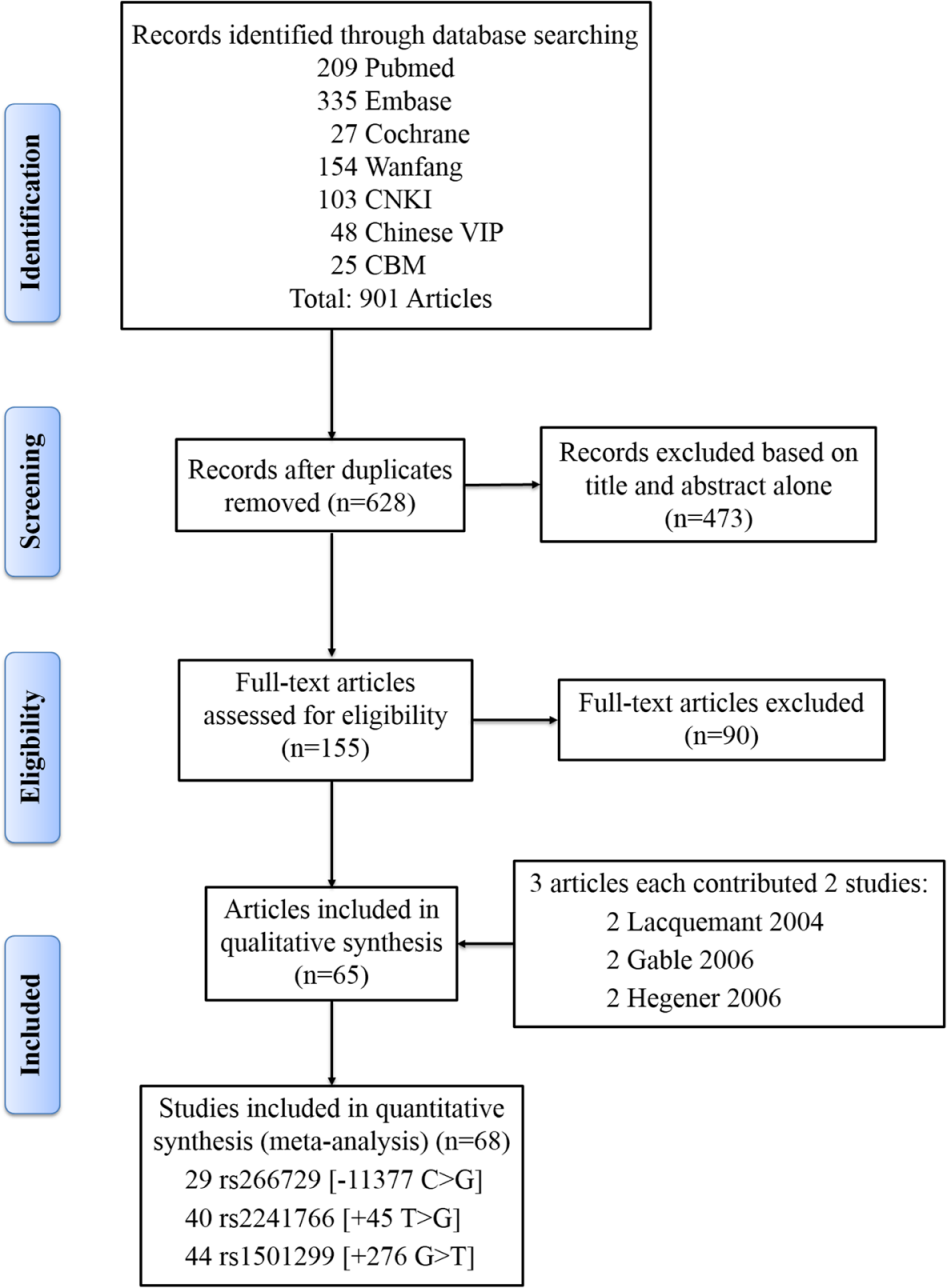
Flow diagram showing details of results of databases searched exclusion and inclusion of studies/articles in the meta-analysis. CNKI: Chinese National Knowledge Infrastructure; CBM: Chinese BioMedical Literature on Disc

This meta-analysis included 68 studies from 65 articles after literature search and critical screening, as described in methods (Fig. 1). Meta-analysis of the rs266729 (− 11,377 C > G), rs2241766 (+ 45 T > G), and rs1501299 (+ 276 G > T) variants included 29, 40, and 44 studies, respectively. We summarize the characteristics of each primary study in Table 1. Detailed characteristics of those studies are further presented in Additional file 1: Tables S1, S2, and S3, respectively. Overall, this meta-analysis included a total of 50,735 subjects (19,106 cases and 31,629 controls).

## Meta-analysis results

### Association between rs266729 (− 11,377 C > G) polymorphism and CVD

**Fig. 2:**
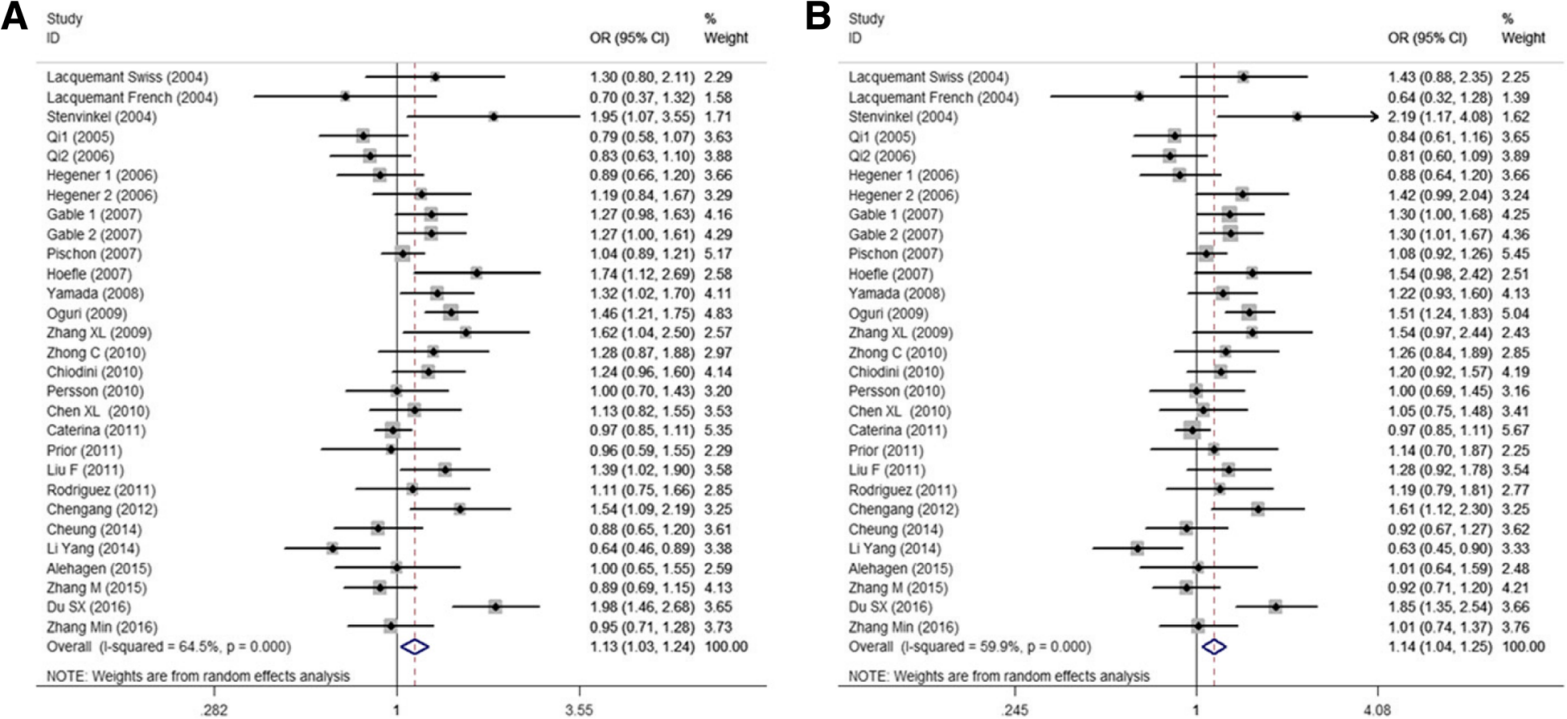
Forest plots of the association between rs266729 polymorphism and CVD risk. (**a**) dominant model; (**b**) heterozygote model

The meta-analysis of the association between rs266729 (− 11,377 C > G) polymorphism and CVD included 29 studies with 29,021 subjects (10,506 cases and 18,515 controls). Significant heterogeneity among studies was observed (*P*_*h*_ < 0.10 or *I*^*2*^ ≥ 50%). Thus, we selected random-effect model, and found that rs266729 polymorphism was associated with the increased risk of CVD in dominant model (GG + GC VS CC: *OR* = 1.129, 95% *CI* = 1.028–1.239, *P* = 0.011) and in heterozygote model (GC VS CC: *OR* = 1.141, 95% *CI* = 1.041–1.250, *P* = 0.005) (Table 2, Fig. 2).

**Table 2:** Overall and subgroup meta-analysis of the association between *ADIPOQ* rs266729, −11,377 C > G polymorphisms and CVD

Categories	*n*	Sample size	G VS C			GG + GC VS CC			GG VS GC + CC			GC VS CC			GG VS CC		
		Case/Control	*OR* (95% *CI*)	*P*	*I*^*2*(^%)/*Ph*	*OR* (95% *CI*)	*P*	*I*^*2*(^%)/*Ph*	*OR* (95% *CI*)	*P*	*I*^*2*(^%)/*Ph*	*OR* (95% *CI*)	*P*	*I*^*2*(^%)/*Ph*	*OR* (95% *CI*)	*P*	*I*^*2*(^%)/*Ph*
Overall	29	10,506/18,515	1.079 (1.000, 1.165)	0.051	65.8/0.000	1.129 (1.028, 1.239)	**0.011**	64.5/0.000	0.989 (0.838, 1.168)	0.898	48.5/0.002	1.141 (1.041, 1.250)	**0.005**	59.9/0.000	1.037 (0.867, 1.239)	0.692	53.4/0.000
Population																	
European	17	6,355/11,666	1.022 (0.948, 1.102)	0.564	37.6/0.060	1.071 (0.974, 1.178)	0.158	40.8/0.041	0.879 (0.714, 1.082)	0.224	40.0/0.045	1.102 (0.995, 1.220)	0.062	43.5/0.029	0.908 (0.739, 1.116)	0.360	36.9/0.064
East Asian	12	4,151/6,849	1.154 (1.000, 1.332)	0.051	76.8/0.000	1.198 (1.006, 1.427)	**0.043**	75.7/0.000	1.149 (0.887, 1.487)	0.293	52.5/0.017	1.184 (1.002, 1.398)	**0.048**	70.7/0.000	1.231 (0.919, 1.650)	0.164	61.4/0.003
Genotyping																	
PCR-RFLP	8	2,382/4,976	1.186 (0.978, 1.438)	0.083	77.5/0.000	1.276 (1.014, 1.607)	**0.038**	75.4/0.000	1.162 (0.813, 1.661)	0.411	53.5/0.035	1.282 (1.032, 1.592)	**0.025**	69.7/0.002	1.285 (0.859, 1.922)	0.223	61.3/0.011
TaqMan	12	3,910/6,312	1.031 (0.935, 1.137)	0.544	45.3/0.044	1.054 (0.948, 1.173)	0.331	30.7/0.146	0.951 (0.720, 1.256)	0.721	53.5/0.014	1.064 (0.960, 1.180)	0.236	20.6/0.242	0.973 (0.735, 1.288)	0.849	52.2/0.018
Others	9	4,214/7,227	1.045 (0.921, 1.186)	0.493	63.6/0.005	1.095 (0.926, 1.296)	0.289	68.7/0.001	0.923 (0.711, 1.197)	0.545	39.6/0.103	1.121 (0.941, 1.336)	0.201	68.7/0.001	0.949 (0.717, 1.255)	0.713	44.9/0.069
Sample size																	
< 1000	21	5,048/6,708	1.065 (0.952, 1.192)	0.270	67.9/0.000	1.114 (0.973, 1.276)	0.119	65.9/0.000	0.955 (0.744, 1.228)	0.722	53.4/0.002	1.128 (0.988, 1.287)	0.075	60.9/0.000	0.992 (0.759, 1.298)	0.956	57.6/0.001
≥ 1000	8	5,458/11,807	1.108 (1.004, 1.222)	**0.042**	63.4/0.008	1.162 (1.026, 1.315)	**0.018**	64.6/0.006	1.017 (0.835, 1.240)	0.864	38.6/0.122	1.172 (1.035, 1.326)	**0.012**	61.5/0.011	1.087 (0.877, 1.347)	0.445	45.4/0.077
Quality score																	
< 10	16	3,489/5,128	1.152 (1.007, 1.318)	**0.040**	68.0/0.000	1.211 (1.032, 1.420)	**0.019**	64.6/0.000	1.147 (0.861, 1.528)	0.348	49.6/0.013	1.207 (1.036, 1.406)	**0.016**	57.8/0.002	1.215 (0.888, 1.664)	0.224	55.9/0.003
≥ 10	13	7,017/13,387	1.019 (0.940, 1.105)	0.646	55.5/0.008	1.062 (0.954, 1.182)	0.271	61.3/0.002	0.883 (0.744, 1.048)	0.155	31.5/0.131	1.089 (0.974, 1.219)	0.135	61.8/0.002	0.915 (0.767, 1.093)	0.327	33.5/0.115

*n* study numbers, Bold values represent statistically significant findings

Based on population, genotyping method, sample size, and quality score, we performed subgroup analyses. On the basis of population, rs266729 polymorphism was associated with the increased risk of CVD under dominant model (GG + GC VS CC: *OR* = 1.198, 95% *CI* = 1.006–1.427, *P* = 0.043) and under heterozygote model (GC VS CC: *OR* = 1.184, 95% *CI* = 1.002–1.398, *P* = 0.048) in East Asian. On the basis of genotyping methods, a significant risk association between rs266729 polymorphism and CVD was found when genotyping was performed using PCR-RFLP method under dominant model (GG + GC VS CC: *OR* = 1.276, 95% *CI* = 1.014–1.607, *P* = 0.038) and under heterozygote model (GC VS CC: *OR* = 1.282, 95% *CI* = 1.032–1.592, *P* = 0.025). On the basis of sample size or quality score, we found that rs266729 polymorphism was associated with the increased risk of CVD under allelic, dominant, and heterozygote models (all *OR* > 1 and *P* < 0.05), after pooled the *ORs* by the subgroups of sample size ≥ 1000 or quality score ≤ 10 (Table 2).

### Association between rs2241766 (+ 45 T > G) polymorphism and CVD

**Fig. 3:**
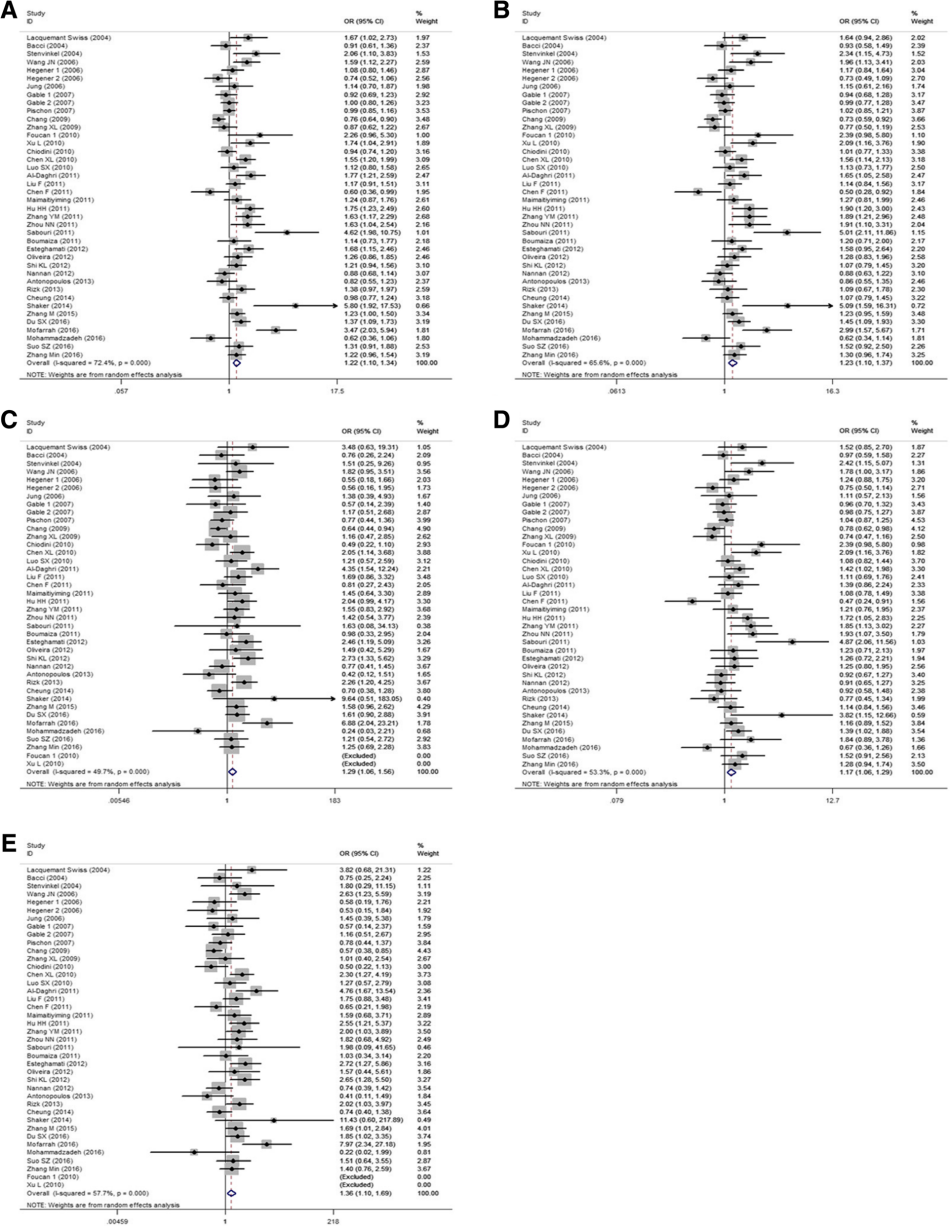
Forest plots of the association between rs2241766 polymorphism and CVD risk. (**a**) allelic model; (**b**) dominant model; (**c**) recessive model; (**d**) heterozygote model; (**e**) homozygote model

The meta-analysis of the association between rs2241766 (+ 45 T > G) polymorphism and CVD included 40 studies with 25,548 subjects (10,746 cases and 14,802 controls). Using inverse-variance weighted random effect model (*P*_*h*_ < 0.10 or *I*^*2*^ ≥ 50%), we found that rs2241766 polymorphism was associated with the increased risk of CVD in the five genetic models (allelic, dominant, recessive, heterozygote, and homozygote) (all *OR* > 1 and *P* < 0.05) (Table 3, Fig. 3).

**Table 3:** Overall and subgroup meta-analysis of the association between *ADIPOQ* rs2241766, +45 T > G polymorphisms and CVD

Categories	*n*	Sample size	G VS T			GG + GT VS TT			GG VS GT + TT			GT VS TT			GG VS TT		
		Case/Control	*OR* (95% *CI*)	*P*	*I*^*2*(^%)/*Ph*	*OR* (95% *CI*)	*P*	*I*^*2*(^%)/*Ph*	*OR* (95% *CI*)	*P*	*I*^*2*(^%)/*Ph*	*OR* (95% *CI*)	*P*	*I*^*2*(^%)/*Ph*	*OR* (95% *CI*)	*P*	*I*^*2*(^%)/*Ph*
Overall	40	10,746/14,802	1.216 (1.102, 1.343)	**< 0.001**	72.4/0.000	1.229 (1.103, 1.369)	**< 0.001**	65.6/0.000	1.286 (1.061, 1.560)	**0.011**	49.7/0.000	1.172 (1.063, 1.292)	**0.001**	53.3/0.000	1.361 (1.095, 1.690)	**0.005**	57.7/0.000
Population																	
European	12	4,452/7,255	1.067 (0.918, 1.242)	0.398	60.4/0.003	1.105 (0.937, 1.303)	0.238	58.3/0.006	0.779 (0.576, 1.055)	0.106	0.0/0.663	1.123 (0.956, 1.319)	0.157	53.0/0.015	0.792 (0.584, 1.073)	0.132	0.0/0.585
East Asian	20	5,305/6,505	1.194 (1.057, 1.348)	**0.004**	70.5/0.000	1.225 (1.057, 1.420)	**0.007**	67.3/0.000	1.315 (1.068, 1.618)	**0.010**	43.1/0.024	1.180 (1.029, 1.353)	**0.018**	58.1/0.001	1.431 (1.112, 1.842)	**0.005**	58.6/0.001
West Asian	5	660/719	1.550 (1.002, 2.396)	**0.049**	80.8/0.000	1.392 (0.893, 2.170)	0.145	71.3/0.007	2.715 (1.452, 5.079)	**0.002**	50.2/0.091	1.099 (0.779, 1.549)	0.591	43.2/0.134	2.767 (1.347, 5.683)	**0.006**	59.2/0.044
African	3	329/323	2.200 (0.890, 5.437)	0.088	75.3/0.017	2.148 (0.952, 4.844)	0.066	65.2/0.056	2.010 (0.251, 16.080)	0.511	50.8/0.154	1.919 (0.998, 3.688)	0.051	45.1/0.162	2.295 (0.250, 21.058)	0.463	55.2/0.135
Genotyping																	
PCR-RFLP	20	4,814/6,319	1.242 (1.055, 1.462)	**0.009**	77.1/0.000	1.279 (1.057, 1.548)	**0.012**	74.4/0.000	1.335 (1.034, 1.722)	**0.027**	40.5/0.035	1.221 (1.023, 1.458)	**0.027**	67.0/0.000	1.442 (1.054, 1.975)	**0.022**	57.1/0.001
TaqMan	7	2,616/3,715	1.087 (0.895, 1.320)	0.400	64.8/0.009	1.118 (0.920, 1.357)	0.262	53.9/0.043	0.872 (0.513, 1.482)	0.614	56.9/0.041	1.123 (0.951, 1.326)	0.172	34.9/0.162	0.896 (0.506, 1.588)	0.708	62.2/0.021
Other	13	3,316/4,768	1.263 (1.075, 1.485)	**0.005**	68.7/0.000	1.238 (1.056, 1.452)	**0.009**	51.4/0.016	1.453 (1.021, 2.066)	**0.038**	56.7/0.006	1.150 (1.004, 1.317)	**0.044**	27.6/0.166	1.522 (1.056, 2.193)	**0.024**	57.7/0.005
Sample size																	
< 1000	34	7,651/6,381	1.298 (1.164, 1.448)	**< 0.001**	66.6/0.000	1.317 (1.163, 1.492)	**< 0.001**	61.1/0.000	1.512 (1.264, 1.809)	**< 0.001**	25.5/0.096	1.239 (1.102, 1.393)	**< 0.001**	51.4/0.000	1.620 (1.324, 1.981)	**< 0.001**	35.9/0.024
≥ 1000	6	3,095/8,421	0.920 (0.834, 1.015)	0.097	23.9/0.255	0.945 (0.841, 1.062)	0.344	25.5/0.243	0.690 (0.539, 0.885)	**0.003**	0.0/0.758	0.981 (0.879, 1.094)	0.728	11.3/0.343	0.669 (0.519, 0.862)	**0.002**	0.0/0.661
Quality score																	
< 10	26	5,467/4,951	1.366 (1.176, 1.586)	**< 0.001**	77.0/0.000	1.404 (1.183, 1.667)	**< 0.001**	72.8/0.000	1.529 (1.202, 1.944)	**0.001**	46.0/0.008	1.314 (1.121, 1.539)	**0.001**	64.4/0.000	1.692 (1.274, 2.248)	**< 0.001**	58.4/0.000
≥ 10	14	5,279/9,851	1.036 (0.944, 1.139)	0.455	37.3/0.079	1.038 (0.955, 1.128)	0.376	0.0/0.575	0.978 (0.719, 1.331)	0.887	49.9/0.017	1.043 (0.956, 1.137)	0.343	0.0/0.818	0.985 (0.725, 1.340)	0.925	47.5/0.025

*n* study numbers; Bold values represent statistically significant findings

Subgroup analyses were stratified by population, genotyping method, sample size, and quality score. Firstly, on the basis of population, rs2241766 polymorphism was associated with the increased risk of CVD under the five dominant models in East Asian and under allelic, recessive, and homozygote models in West Asian (all *OR* > 1 and *P* < 0.05). Secondly, on the basis of genotyping method, the results that genotyping was done by PCR-RFLP or other methods showed that rs2241766 polymorphism was associated with the increased risk of CVD under five genetic models (all *OR* > 1 and *P* < 0.05). Thirdly, on the basis of sample size, rs2241766 polymorphism was associated with the increased risk of CVD under the five genetic models in the subgroup of sample size ≤1000 (all *OR* > 1 and *P* < 0.05), but was associated with the decreased risk of CVD in the subgroup of sample size ≥1000 under recessive model (GG VS GT + TT: *OR* = 0.696, 95% *CI* = 0.539–0.885, *P* = 0.003) and under homozygote model (GG VS TT: *OR* = 0.669, 95% *CI* = 0.519–0.862, *P* = 0.002). Finally, on the basis of quality score, when we pooled the *ORs* by the subgroups of quality score ≤ 10, we found that rs2241766 polymorphism was associated with the increased risk of CVD under the five genetic models (all *OR* > 1 and *P* < 0.05) (Table 3).

### Association between rs1501299 (+ 276 G > T) polymorphism and CVD

The meta-analysis of the association between rs1501299 (+ 276 G > T) polymorphism and CVD included 44 studies with 37,371 subjects (12,852 cases and 24,519 controls). Using the inverse-variance weighted random effect model (*P*_*h*_ < 0.10 or *I*^*2*^ ≥ 50%), we found that there was no association between rs1501299 polymorphism and CVD in the five genetic models (all *P* > 0.05) (Table 4).

**Table 4:** Overall and subgroup meta-analysis of the association between *ADIPOQ* rs1501299, +276 G > T polymorphism and CVD

Categories	n	Sample size	T VS G			TT + TG VS GG			TT VS TG + GG			TG VS GG			TT VS GG		
		Case/Control	*OR* (95%*CI*)	*P*	*I*^*2*(^%)/*Ph*	*OR* (95%*CI*)	*P*	*I*^*2*(^%)/*Ph*	*OR* (95%*CI*)	*P*	*I*^*2*(^%)/*Ph*	*OR* (95%*CI*)	*P*	*I*^*2*(^%)/*Ph*	*OR* (95%*CI*)	*P*	*I*^*2*(^%)/*Ph*
Overall	44	12,852/24,519	0.956 (0.893, 1.023)	0.189	64.7/0.000	0.967 (0.890, 1.051)	0.431	60.6/0.000	0.899 (0.797, 1.015)	0.086	42.0/0.002	0.987 (0.913, 1.066)	0.737	49.2/0.000	0.886 (0.766, 1.025)	0.104	55.5/0.000
Population																	
European	18	7,002/11,337	0.957 (0.901, 1.016)	0.146	16.2/0.260	0.967 (0.896, 1.043)	0.380	16.1/0.262	0.851 (0.717, 1.011)	0.066	35.2/0.070	0.988 (0.909, 1.073)	0.773	21.3/0.201	0.854 (0.722, 1.012)	0.068	30.2/0.110
East Asian	20	5,107/12,291	0.966 (0.849, 1.098)	0.594	77.5/0.000	0.977 (0.834, 1.145)	0.776	74.4/0.000	0.945 (0.778, 1.149)	0.572	52.2/0.004	0.988 (0.858, 1.138)	0.867	64.0/0.000	0.940 (0.726, 1.217)	0.638	69.0/0.000
West Asian	4	479/645	0.973 (0.643, 1.473)	0.897	79.6/0.002	0.960 (0.564, 1.635)	0.880	77.8/0.004	0.999 (0.578, 1.727)	0.997	41.9/0.160	0.952 (0.587, 1.546)	0.843	70.2/0.018	0.986 (0.477, 2.040)	0.970	62.1/0.048
African	2	264/246	0.848 (0.629, 1.143)	0.278	11.8/0.287	0.856 (0.583, 1.257)	0.428	0.0/0.490	0.724 (0.415, 1.266)	0.258	7.8/0.298	0.927 (0.614, 1.400)	0.719	0.0/0.725	0.700 (0.374, 1.312)	0.266	14.7/0.279
Genotyping																	
PCR-RFLP	14	3,359/5,817	0.970 (0.833, 1.128)	0.688	74.2/0.000	0.997 (0.825, 1.206)	0.978	70.6/0.000	0.881 (0.684, 1.136)	0.329	55.3/0.006	1.051 (0.858, 1.202)	0.861	58.4/0.003	0.901 (0.648, 1.253)	0.535	68.4/0.000
TaqMan	13	3,666/6,001	0.977 (0.869, 1.099)	0.701	61.3/0.002	0.987 (0.854, 1.140)	0.859	58.0/0.005	0.970 (0.791, 1.189)	0.771	30.1/0.144	1.001 (0.874, 1.146)	0.994	47.8/0.028	0.956 (0.749, 1.221)	0.718	46.8/0.032
Others	17	5,827/12,701	0.930 (0.841, 1.029)	0.159	59.7/0.001	0.935 (0.827, 1.058)	0.287	55.1/0.003	0.866 (0.715, 1.048)	0.140	40.9/0.041	0.959 (0.852, 1.079)	0.484	46.3/0.019	0.841 (0.678, 1.044)	0.117	49.6/0.011
Sample size																	
< 1000	36	8,167/9,201	0.945 (0.868, 1.029)	0.191	64.8/0.000	0.959 (0.864, 1.065)	0.438	60.2/0.000	0.876 (0.756, 1.016)	0.079	41.8/0.005	0.985 (0.895, 1.085)	0.758	47.8/0.001	0.865 (0.722, 1.036)	0.116	56.0/0.000
≥ 1000	8	4,685/15,318	0.984 (0.877, 1.104)	0.784	68.6/0.002	0.985 (0.855, 1.134)	0.831	66.7/0.004	0.968 (0.784, 1.195)	0.762	44.8/0.080	0.987 (0.863, 1.129)	0.853	59.7/0.015	0.955 (0.748, 1.219)	0.711	56.3/0.025
Quality score																	
< 10	24	4,690/5,424	0.954 (0.848, 1.074)	0.438	69.1/0.000	0.976 (0.842, 1.132)	0.752	65.3/0.000	0.879 (0.725, 1.065)	0.189	41.7/0.018	1.002 (0.876, 1.145)	0.981	52.8/0.001	0.876 (0.683, 1.122)	0.294	59.3/0.000
≥ 10	20	8,162/19,095	0.959 (0.886, 1.038/)	0.298	60.0/0.000	0.963 (0.875, 1.060)	0.442	55.3/0.002	0.915 (0.782, 1.072)	0.273	44.7/0.017	0.976 (0.890, 1.070)	0.599	46.3/0.013	0.902 (0.756, 1.075)	0.250	52.2/0.004

In the subgroup analysis, no significant association was found between rs1501299 polymorphism and CVD risk under the five genetic models in any subgroup (all *P* > 0.05) (Table 4).

### Heterogeneity analysis

In this meta-analysis, meta-regression was used to investigate the source of heterogeneity by year, population, genotyping method, sample size, and quality score. We found that sample size (allelic model: *P* = 0.019; dominant model: *P* = 0.032; recessive model: *P* < 0.001; and homozygote model: *P* < 0.001) and quality score (allelic model: *P* = 0.035; dominant model: *P* = 0.032; recessive model: *P* < 0.001; and homozygote model: *P* < 0.001) contributed to the observed heterogeneity across all the studies of the association between rs2241766 polymorphisms and CVD risk. However, in the meta-analysis of the associations between rs266729/rs1501299 polymorphisms and CVD risk, we did not identify the source of heterogeneity (all *P* > 0.05) (Additional file 2: Table S4).

### Publication bias and sensitivity analysis

**Fig. 4:**
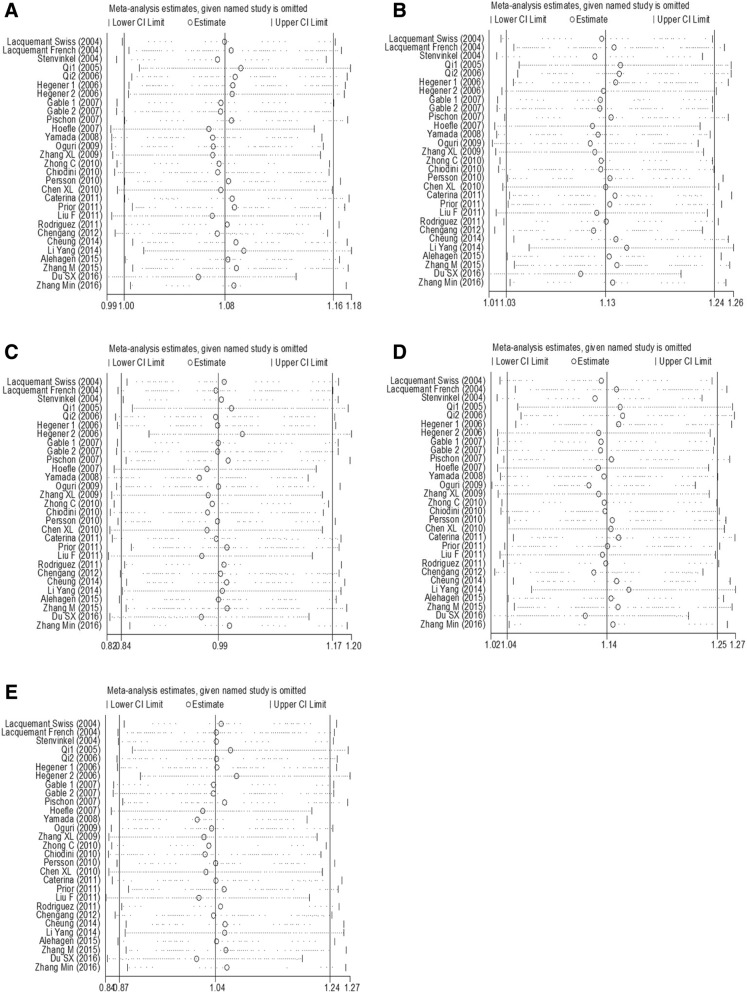
Sensitivity analyses of the association between rs266729 polymorphism and CVD risk. (**a**) allelic model; (**b**) dominant model; (**c**) recessive model; (**d**) heterozygote model; (**e**) homozygote model

**Fig. 5:**
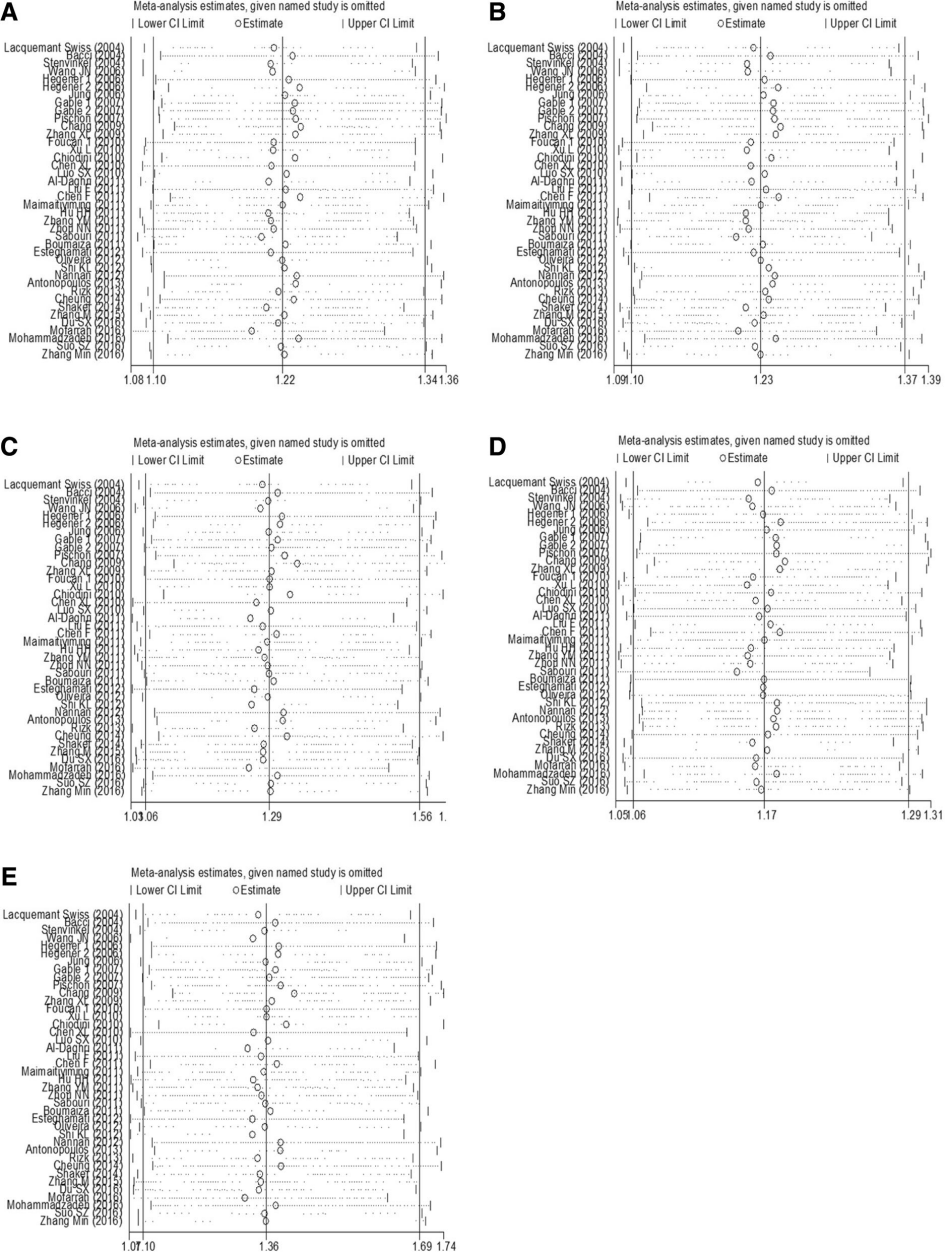
Sensitivity analyses of the association between rs2241766 polymorphism and CVD risk. (**a**) allelic model; (**b**) dominant model; (**c**) recessive model; (**d**) heterozygote model; (**e**) homozygote model

**Fig. 6:**
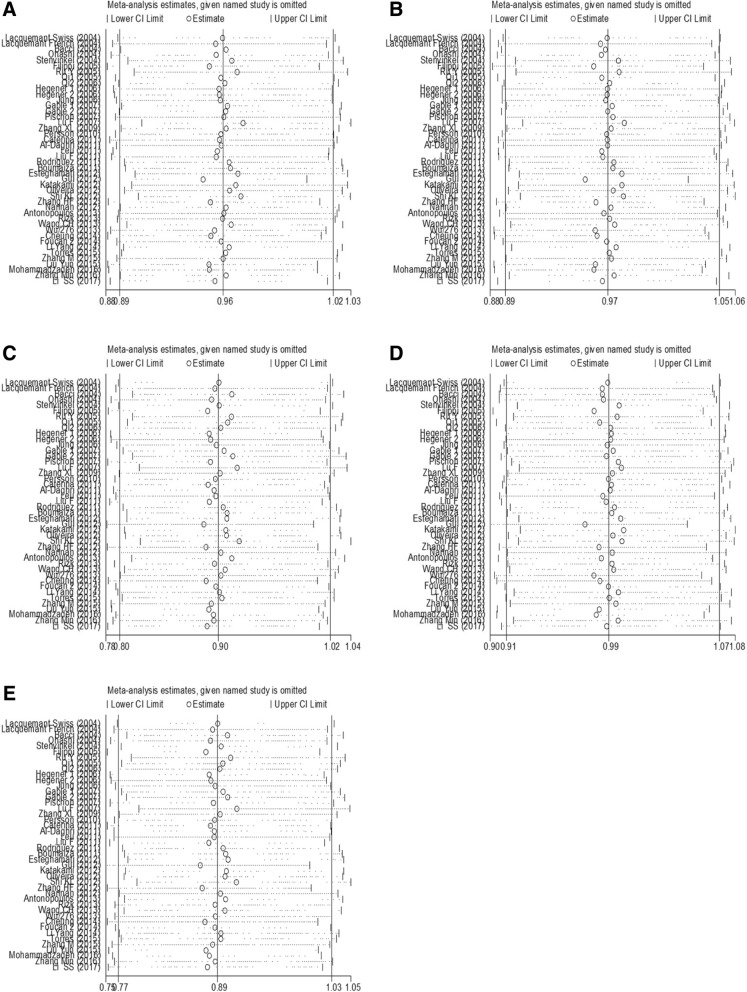
Sensitivity analyses of the association between rs1501299 polymorphism and CVD risk. (**a**) allelic model; (**b**) dominant model; (**c**) recessive model; (**d**) heterozygote model; (**e**) homozygote model

Publication bias was measured by funnel plots and quantified by Begg’s and Egger’s tests. No publication bias was found among the studies regarding the association between rs266729 polymorphisms and CVD risk (all *P* > 0.05). Publication biases were found in analyses of the associations between rs2241766 polymorphisms and CVD risk (allelic model: *P*_Egger_ = 0.001, *P*_Begg_ = 0.031; dominant model: *P*_Egger_ = 0.001, *P*_Begg_ = 0.003; and heterozygote mode: *P*_Egger_ = 0.003, *P*_Begg_ = 0.003), and between rs1501299 polymorphisms and CVD risk (recessive model: *P*_Egger_ = 0.031, *P*_Begg_ = 0.035) (Table 5 and Additional file 3: Figures S1, S2, and S3). Sensitivity analyses showed that this meta-analysis was relatively stable and credible (Figs. 4, 5, and 6).

**Table 5:** Publication bias assessment of this meta-analysis

SNPs	Genetic model	Egger’s test		Begg’s test	
		t-value	*P*	z-value	*P*
rs266729	Allelic model	0.60	0.552	0.47	0.639
	Dominant model	0.77	0.451	0.62	0.536
	Recessive model	−0.67	0.507	0.92	0.358
	Heterozygote model	0.79	0.435	0.81	0.420
	Homozygote model	−0.45	0.658	0.73	0.464
rs2241766	Allelic model	3.52	0.001	2.16	0.031
	Dominant model	3.63	0.001	2.99	0.003
	Recessive model	0.72	0.476	0.40	0.687
	Heterozygote model	3.17	0.003	2.97	0.003
	Homozygote model	0.88	0.383	0.33	0.744
rs1501299	Allelic model	−0.80	0.427	0.96	0.337
	Dominant model	0.09	0.930	0.13	0.895
	Recessive model	−2.24	0.031	2.11	0.035
	Heterozygote model	0.60	0.549	0.11	0.911
	Homozygote model	−1.45	0.155	1.49	0.137

### TSA

**Fig. 7:**
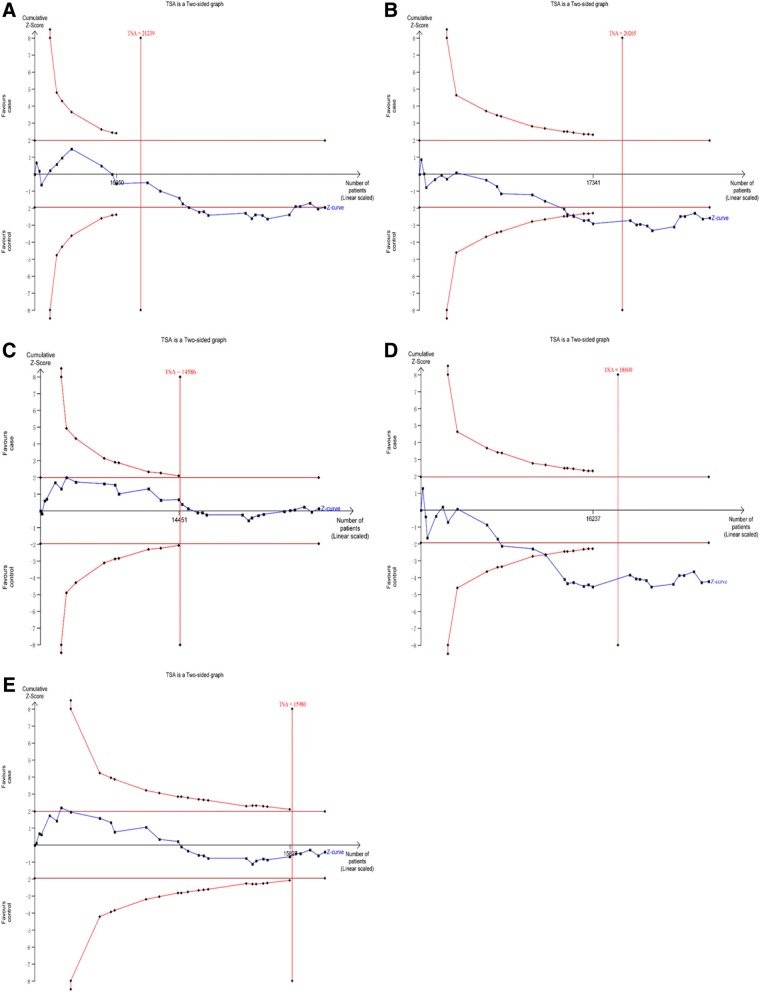
Trial sequential analysis of the association between rs266729 and CVD risk. (**a**) allelic model; (**b**) dominant model; (**c**) recessive model; (**d**) heterozygote model; (**e**) homozygote model

**Fig. 8:**
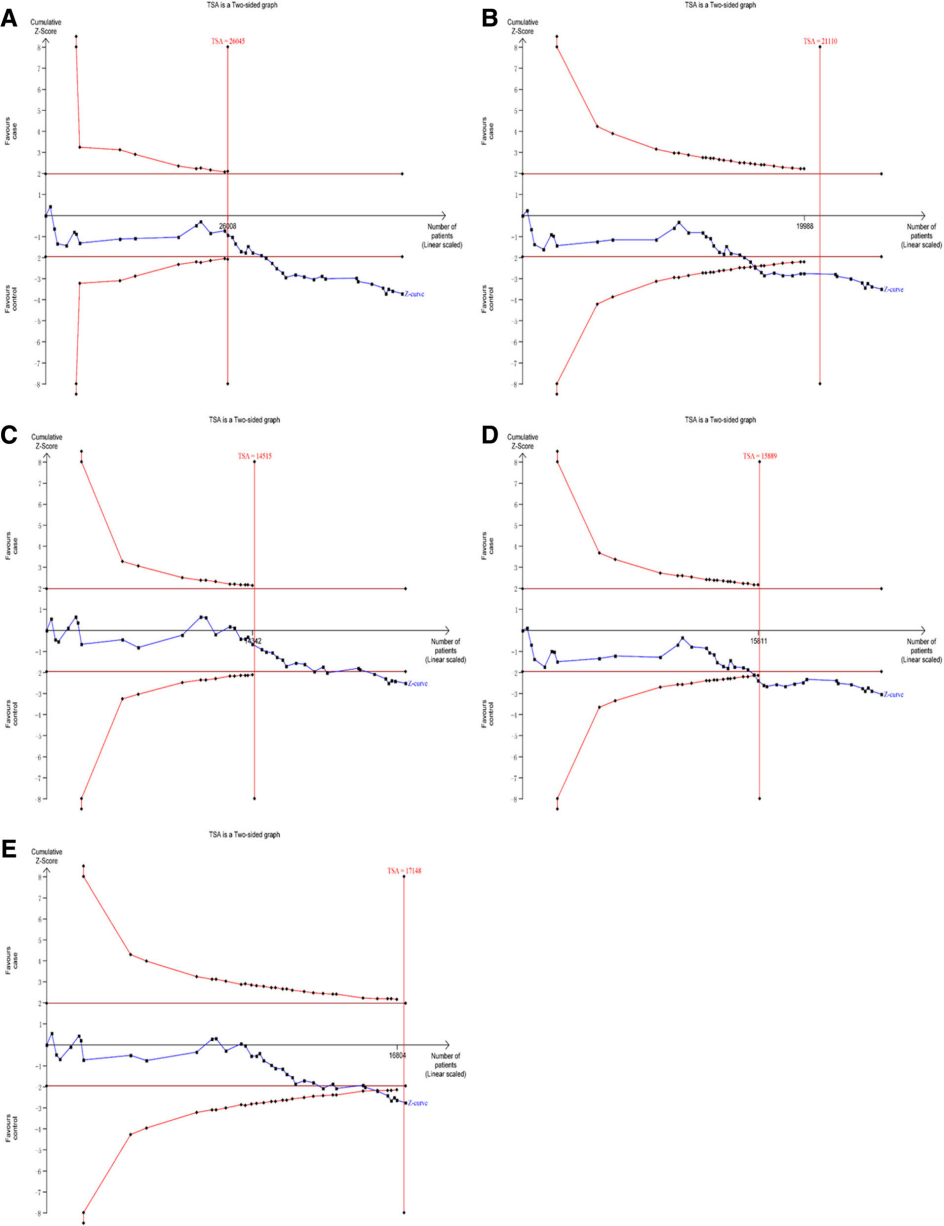
Trial sequential analysis of the association between rs2241766 and CVD risk. (**a**) allelic model; (**b**) dominant model; (**c**) recessive model; (**d**) heterozygote model; (**e**) homozygote model

**Fig. 9:**
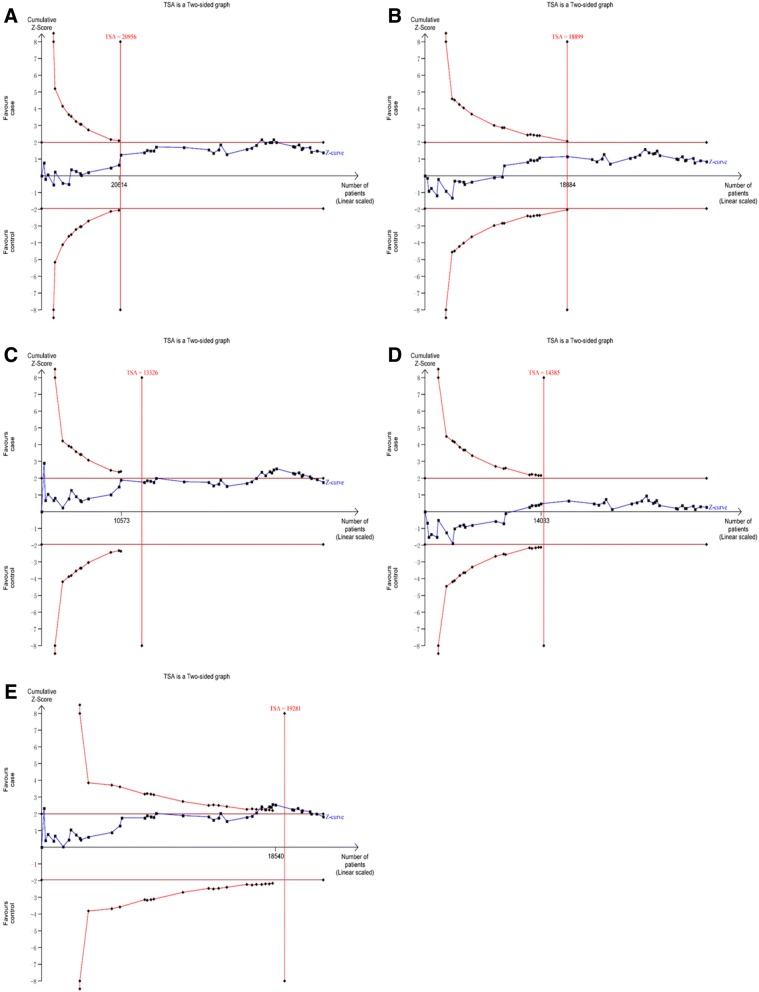
Trial sequential analysis of the association between rs1501299 and CVD risk. (**a**) allelic model; (**b**) dominant model; (**c**) recessive model; (**d**) heterozygote model; (**e**) homozygote model

In the TSA of rs266729 and CVD, the Z-curve crossed trial sequential monitoring boundary and the sample size reached RIS in dominant and heterozygote models (Fig. 7). In allelic, recessive, and homozygote models, the sample size also reached RIS, although the Z-curve did not cross trial sequential monitoring boundary (Fig. 7). In the TSA of rs2241766/rs1501299 and CVD, the sample size reached RIS in the five genetic models (Figs. 8 and 9). Thus, concrete conclusions were reached and further studies were not required.

## Discussion

In this meta-analysis, we collected up-to-date information (July 1st, 2017) to investigate the association between *ADIPOQ* SNPs and the risk of CVD. Our results demonstrate that rs266729 and rs2241766 variants of *ADIPOQ* are associated with the increased risk of CVD, but rs1501299 is not associated with CVD risk.

In view of the association between rs266729 and CVD risk, Yang et al. (2012) [60], Zhou et al. (2012) [61], and Zhang et al. (2012) [50] performed meta-analyses. Yang et al. reported that rs266729 is associated with the increased risk of CAD in allelic and dominant models [60]. Zhou et al. found the same association in overall population, Europeans, and East Asian in allelic, dominant and heterozygote models [61]. Zhang et al. also revealed that rs266729 is associated with the increased risk of CAD in overall population and East Asian in allelic model [50]. Our results further identified that rs266729 is associated with the increased risk of CVD in overall population and East Asian in dominant and heterozygote models. In addition, our results revealed that the significant association in studies on the basis of PCR-RFLP method, indicating that different genotyping method may result in different statistical results.

The association between rs2241766 and CVD risk also has been the subject of meta-analysis [60–64]. These studies are inconsistent. Yang et al. found no significant association between rs2241766 and CAD risk [60]. Zhang et al. found no overall significant risk association between CHD and rs2241766 in Han Chinese population [62]. Zhou et al. reported that rs2241766 is associated with the decreased risk of CVD in recessive and homozygote models, and the decreased risk of CVD in East Asian in allelic, dominant, recessive, and homozygote models [61]. Zhou et al. performed a meta-analysis of the association between rs2241766 and CVD risk in allelic model, and they found that rs2241766 is associated with the increased risk of CVD [63]. In our meta-analysis, we found that rs2241766 is associated with the increased risk of CVD in overall population and East Asian in all the five genetic models, and in West Asian in allelic, recessive, and homozygote models. Our findings is in agreement with the results of Zhou et al., but is in disagreement with the results of Yang et al., Zhang et al., and Zhou et al.

With regard to the association between 1,501,299 and CVD, the results are also conflicting [50, 60, 61]. *Zhou* et al. revealed no significant association between rs1501299 polymorphism with CAD susceptibility [61]. Qi et al. reported the extremely large decrease in CVD risk associated with rs1501299 polymorphism in diabetic patients [24]. Zhang et al. reported only the weak protective effect of the rs1501299 variant against CVD in general study subjects [50]. The meta-analysis by Zhao et al. revealed that rs1501299 polymorphism may play a protective role for CAD among patients with T2DM [22]. In comparison, our results revealed no significant association.

Different genetic admixture and environmental factors among human populations, which tend to explain ethnic background, strongly modulate the effects of *ADIPOQ* polymorphisms on adiponectin levels [65, 66]. Studies have reported that low levels of adiponectin (hypoadipoectinemia) correlate with the risk of CVD, and high levels of adiponectin protect against this disease [6–11]. These conflicting results of associations between the *ADIPOQ* polymorphism and CVD risk may be due to differences in publication bias, sample size, or insufficient statistical power. In addition, evidences have showed that studies which deviate from HWE in controls may reflect the presence of genotyping errors, population stratification, and selection bias in the controls (or without representation of studied sample). Thus, including those studies may decrease the quality of a meta-analysis or generate inconsistent results [67].

Heterogeneity across all the studies of the associations should be noted because it may potentially affect the strengths of the present meta-analysis. We, thus, used random effect model. Our results showed that sample size and quality score are the factors of heterogeneity across all studies of association between rs2241766 polymorphisms and CVD, but no factors contribute the heterogeneity across all studies of association between rs266729/rs1501299 polymorphisms and CVD. However, heterogeneity was still high in the subgroup analysis of the two factors. For these reasons, heterogeneity might be explained by other confounding factors, such as gene-gene interaction and gene-environment interaction.

Our meta-analysis has some limitations. Firstly, significant publication bias was found in the analysis of rs2241766 (under allelic, dominant, and heterozygote models) and rs1501299 (under recessive model). Secondly, our meta-analysis mainly included Europeans and Asians with only few other races, thus limiting our power to generalize our findings in other races. Finally, our results might be affected by the potential weaknesses of genetic association studies, such as phenotype misclassifications, genotyping error, population stratification, gene-environment or gene-gene interactions, and selective reporting biases [68, 69].

Despite the limitations highlighted above, our meta-analysis also had some strength. Firstly, we searched extensively and investigated more studies and more participants than any other meta-analyses performed on the association between *ADIPOQ* variant and CVD, which give our study more statistical power to draw valid conclusion on this issue. Secondly, sensitivity analysis showed that the results of our meta-analysis are stable and robust. Thirdly, the evidence of our results are sufficient to reach concrete conclusions, which were proved by TSA for the first time. We strongly believe our findings will help settle some of the controversies surrounding the *ADIPOQ*-CVD association research.

## Conclusions

Our meta-analysis found significant increased CVD risk is associated with rs266729 and rs2241766, but not associated with rs1501299. Investigating gene–gene and gene–environment interactions is needed to give more insight into the genetic association between *ADIPOQ* variants and CVD.

## Additional files

Additional file 1: Summary of three SNPs characteristics. (DOCX 48 kb)

Additional file 2: Meta-regression results of the association between the SNPs and CVD risk. (DOCX 59 kb)

Additional file 3: Funnel plots of three SNPs for publication bias. (DOCX 1250 kb)

Additional file 4: Additional references. (DOCX 22 kb)

## Abbreviations

CAD: Coronary artery disease
CHD: Coronary heart disease
CVD: Cardiovascular disease
HWE: Hardy–Weinberg equilibrium
MI: Myocardial infarction
RIS: Required information size
SNPs: Single-nucleotide polymorphisms
T2DM: Type 2 diabetic mellitus
TSA: Trial sequential analysis
