# Supplementary material for: Associations between three common single nucleotide polymorphisms (rs266729, rs2241766, and rs1501299) of ADIPOQ and cardiovascular disease: a meta-analysis
Source: Lipids Health Dis. 2018 May 28;17:126. doi: 10.1186/s12944-018-0767-8 (PMC5972450; doi:10.1186/s12944-018-0767-8)
Supplement: Supplementary file 1 — Summary of three SNPs characteristics. (DOCX 48 kb) [file 12944_2018_767_MOESM1_ESM.docx]

| Table S1 Summary of SNP rs266729, −11377 CG characteristics | | | | | | | | | | | | | |
| --- | --- | --- | --- | --- | --- | --- | --- | --- | --- | --- | --- | --- | --- |
| Study | ID | Year | Country | Population | Total | Case | Control | CaseCC | CaseCG | CaseGG | ControlCC | ControlCG | ControlGG |
| [Lacquemant Swiss](file:///F:\百度云同步盘\研究僧\卡奴meta分析\数据\表格%20最终%20终删掉outcome.xlsx#RANGE!_ENREF_19)^70^ | 1 | 2004 | Switzerland | European | 287 | 106 | 181 | 57 | 48 | 1 | 109 | 64 | 8 |
| Lacquemant French^70^ | 2 | 2004 | France | European | 189 | 55 | 134 | 32 | 17 | 6 | 65 | 54 | 13 |
| [Stenvinkel](file:///F:\百度云同步盘\研究僧\卡奴meta分析\数据\表格%20最终%20终删掉outcome.xlsx#RANGE!_ENREF_60)^41^ | 3 | 2004 | America | European | 204 | 63 | 141 | 29 | 31 | 3 | 88 | 43 | 10 |
| [Qi1](file:///F:\百度云同步盘\研究僧\卡奴meta分析\数据\表格%20最终%20终删掉outcome.xlsx#RANGE!_ENREF_22)^74^ | 4 | 2005 | America | European | 852 | 232 | 620 | 138 | 85 | 9 | 333 | 243 | 44 |
| [Qi2](file:///F:\百度云同步盘\研究僧\卡奴meta分析\数据\表格%20最终%20终删掉outcome.xlsx#RANGE!_ENREF_63)^24^ | 5 | 2006 | America | European | 961 | 279 | 682 | 158 | 102 | 19 | 355 | 283 | 44 |
| [Hegener 1](file:///F:\百度云同步盘\研究僧\卡奴meta分析\数据\表格%20最终%20终删掉outcome.xlsx#RANGE!_ENREF_66)^76^ | 6 | 2006 | America | European | 682 | 341 | 341 | 198 | 123 | 20 | 188 | 133 | 20 |
| [Hegener 2](file:///F:\百度云同步盘\研究僧\卡奴meta分析\数据\表格%20最终%20终删掉outcome.xlsx#RANGE!_ENREF_66)^76^ | 7 | 2006 | America | European | 518 | 259 | 259 | 123 | 128 | 8 | 134 | 98 | 27 |
| [Gable 1](file:///F:\百度云同步盘\研究僧\卡奴meta分析\数据\表格%20最终%20终删掉outcome.xlsx#RANGE!_ENREF_64)^77^ | 8 | 2007 | UK | European | 2988 | 266 | 2722 | 129 | 120 | 17 | 1480 | 1063 | 179 |
| [Gable 2](file:///F:\百度云同步盘\研究僧\卡奴meta分析\数据\表格%20最终%20终删掉outcome.xlsx#RANGE!_ENREF_64)^77^ | 9 | 2007 | UK | European | 1094 | 530 | 564 | 278 | 217 | 35 | 329 | 197 | 38 |
| Pischon^42^ | 10 | 2007 | America | European | 3107 | 1036 | 2071 | 574 | 408 | 54 | 1166 | 767 | 138 |
| Hoefle^79^ | 11 | 2007 | Austria | European | 402 | 277 | 125 | 145 | 109 | 23 | 82 | 40 | 3 |
| Yamada^80^ | 12 | 2008 | Japan | East Asian | 1282 | 311 | 971 | 163 | 120 | 28 | 575 | 346 | 50 |
| Oguri^81^ | 13 | 2009 | Japan | East Asian | 1887 | 773 | 1114 | 397 | 336 | 40 | 675 | 379 | 60 |
| Zhang XL^82^ | 14 | 2009 | China | East Asian | 340 | 205 | 135 | 94 | 87 | 24 | 78 | 47 | 10 |
| Zhong C^83^ | 15 | 2010 | China | East Asian | 435 | 198 | 237 | 110 | 72 | 16 | 146 | 76 | 15 |
| Chiodini^29^ | 16 | 2010 | Italy | European | 1006 | 503 | 503 | 295 | 177 | 31 | 321 | 160 | 22 |
| Persson^86^ | 17 | 2010 | Sweden | European | 488 | 244 | 244 | 129 | 100 | 15 | 129 | 100 | 15 |
| Chen XL^87^ | 18 | 2010 | China | East Asian | 621 | 317 | 304 | 174 | 108 | 35 | 176 | 104 | 24 |
| Caterina^89^ | 19 | 2011 | Italy | European | 3710 | 1855 | 1855 | 1076 | 671 | 108 | 1063 | 684 | 108 |
| Prior^91^ | 20 | 2011 | UK | European | 383 | 85 | 298 | 46 | 38 | 1 | 158 | 114 | 26 |
| Liu F^28^ | 21 | 2011 | China | East Asian | 640 | 302 | 338 | 144 | 125 | 33 | 189 | 128 | 21 |
| Rodriguez^93^ | 22 | 2011 | Spain | European | 674 | 119 | 555 | 67 | 46 | 6 | 327 | 188 | 40 |
| Chengang^101^ | 23 | 2012 | China | East Asian | 517 | 267 | 250 | 105 | 147 | 15 | 125 | 109 | 16 |
| Cheung^110^ | 24 | 2014 | China | East Asian | 2191 | 184 | 2007 | 111 | 65 | 8 | 1148 | 729 | 130 |
| Li Yang^111^ | 25 | 2014 | China | East Asian | 599 | 234 | 365 | 145 | 76 | 13 | 186 | 154 | 25 |
| Alehagen^112^ | 26 | 2015 | Sweden | European | 476 | 105 | 371 | 58 | 40 | 7 | 205 | 140 | 26 |
| Zhang M^114^ | 27 | 2015 | China | East Asian | 973 | 561 | 412 | 305 | 228 | 28 | 212 | 172 | 28 |
| Du SX^39^ | 28 | 2016 | China | East Asian | 797 | 493 | 304 | 271 | 189 | 33 | 215 | 81 | 8 |
| Zhang Min^40^ | 29 | 2016 | China | East Asian | 718 | 306 | 412 | 161 | 132 | 13 | 212 | 172 | 28 |

The 70-117 references are listed in Additional file 4

| Table S2 Summary of SNP rs2241766, +45TG characteristics | | | | | | | | | | | | | |
| --- | --- | --- | --- | --- | --- | --- | --- | --- | --- | --- | --- | --- | --- |
| Study | ID | Year | Country | Population | Total | Case | Control | CaseTT | CaseTG | CaseGG | ControlTT | ControlTG | ControlGG |
| [Lacquemant Swiss](file:///F:\百度云同步盘\研究僧\卡奴meta分析\数据\表格%20最终%20终删掉outcome.xlsx#RANGE!_ENREF_19)^70^ | 1 | 2004 | Switzerland | European | 288 | 107 | 181 | 76 | 27 | 4 | 145 | 34 | 2 |
| [Bacci](file:///F:\百度云同步盘\研究僧\卡奴meta分析\数据\表格%20最终%20终删掉outcome.xlsx#RANGE!_ENREF_18)^47^ | 2 | 2004 | Italy | European | 350 | 130 | 220 | 90 | 35 | 5 | 149 | 60 | 11 |
| [Stenvinkel](file:///F:\百度云同步盘\研究僧\卡奴meta分析\数据\表格%20最终%20终删掉outcome.xlsx#RANGE!_ENREF_60)^41^ | 3 | 2004 | America | European | 204 | 63 | 141 | 44 | 17 | 2 | 119 | 19 | 3 |
| [Wang JN](file:///F:\百度云同步盘\研究僧\卡奴meta分析\数据\表格%20最终%20终删掉outcome.xlsx#RANGE!_ENREF_66)^75^ | 4 | 2006 | China | East Asian | 251 | 120 | 131 | 28 | 65 | 27 | 49 | 64 | 18 |
| [Hegener 1](file:///F:\百度云同步盘\研究僧\卡奴meta分析\数据\表格%20最终%20终删掉outcome.xlsx#RANGE!_ENREF_66)^76^ | 5 | 2006 | America | European | 682 | 341 | 341 | 241 | 95 | 5 | 252 | 80 | 9 |
| [Hegener 2](file:///F:\百度云同步盘\研究僧\卡奴meta分析\数据\表格%20最终%20终删掉outcome.xlsx#RANGE!_ENREF_66)^76^ | 6 | 2006 | America | European | 518 | 259 | 259 | 203 | 52 | 4 | 188 | 64 | 7 |
| Jung^43^ | 7 | 2006 | Korea | East Asian | 156 | 88 | 68 | 41 | 40 | 7 | 34 | 30 | 4 |
| [Gable 1](file:///F:\百度云同步盘\研究僧\卡奴meta分析\数据\表格%20最终%20终删掉outcome.xlsx#RANGE!_ENREF_64)^77^ | 8 | 2007 | UK | European | 2864 | 259 | 2605 | 204 | 53 | 2 | 2022 | 548 | 35 |
| [Gable 2](file:///F:\百度云同步盘\研究僧\卡奴meta分析\数据\表格%20最终%20终删掉outcome.xlsx#RANGE!_ENREF_64)^77^ | 9 | 2007 | UK | European | 1089 | 526 | 563 | 360 | 154 | 12 | 384 | 168 | 11 |
| Pischon^42^ | 10 | 2007 | America | European | 3074 | 1023 | 2051 | 775 | 231 | 17 | 1560 | 447 | 44 |
| Chang^46^ | 11 | 2009 | China | East Asian | 1287 | 600 | 687 | 316 | 238 | 46 | 309 | 299 | 79 |
| Zhang XL^82^ | 12 | 2009 | China | East Asian | 340 | 205 | 135 | 106 | 85 | 14 | 61 | 66 | 8 |
| Foucan 1^84^ | 13 | 2010 | France | African | 216 | 57 | 159 | 47 | 10 | 0 | 146 | 13 | 0 |
| Xu L^85^ | 14 | 2010 | China | East Asian | 226 | 153 | 73 | 78 | 75 | 0 | 50 | 23 | 0 |
| Chiodini^29^ | 15 | 2010 | Italy | European | 1006 | 503 | 503 | 358 | 136 | 9 | 359 | 126 | 18 |
| Chen XL^87^ | 16 | 2010 | China | East Asian | 679 | 345 | 334 | 192 | 117 | 36 | 221 | 95 | 18 |
| Luo SX^88^ | 17 | 2010 | China | East Asian | 321 | 221 | 100 | 100 | 99 | 22 | 50 | 41 | 9 |
| Al-Daghri^90^ | 18 | 2011 | Saudi A. | West Asian | 420 | 122 | 298 | 77 | 35 | 10 | 220 | 72 | 6 |
| Liu F^28^ | 19 | 2011 | China | East Asian | 640 | 302 | 338 | 157 | 123 | 22 | 187 | 136 | 15 |
| Chen F^94^ | 20 | 2011 | China | East Asian | 195 | 93 | 102 | 68 | 19 | 6 | 59 | 35 | 8 |
| Maimaitiyiming^95^ | 21 | 2011 | China | East Asian | 320 | 196 | 124 | 91 | 85 | 20 | 65 | 50 | 9 |
| Hu HH^96^ | 22 | 2011 | China | East Asian | 302 | 150 | 152 | 66 | 60 | 24 | 91 | 48 | 13 |
| Zhang YM^97^ | 23 | 2011 | China | East Asian | 316 | 149 | 167 | 63 | 60 | 26 | 97 | 50 | 20 |
| Zhou NN^98^ | 24 | 2011 | China | East Asian | 423 | 358 | 65 | 175 | 145 | 38 | 42 | 18 | 5 |
| Sabouri^99^ | 25 | 2011 | UK | European | 435 | 329 | 106 | 253 | 74 | 2 | 100 | 6 | 0 |
| Boumaiza^100^ | 26 | 2011 | Tunisia | African | 316 | 212 | 104 | 145 | 57 | 10 | 75 | 24 | 5 |
| Esteghamati^48^ | 27 | 2012 | Iran | West Asian | 241 | 114 | 127 | 48 | 41 | 25 | 68 | 46 | 13 |
| Oliveira^44^ | 28 | 2012 | Brazil | European | 603 | 450 | 153 | 323 | 114 | 13 | 117 | 33 | 3 |
| Shi KL^103^ | 29 | 2012 | China | East Asian | 688 | 396 | 292 | 227 | 134 | 35 | 172 | 110 | 10 |
| Nannan^105^ | 30 | 2012 | China | East Asian | 680 | 213 | 467 | 115 | 84 | 14 | 237 | 191 | 39 |
| Antonopoulos^106^ | 31 | 2013 | Greece | European | 594 | 462 | 132 | 359 | 97 | 6 | 99 | 29 | 4 |
| Rizk^107^ | 32 | 2013 | Qatar | West Asian | 264 | 142 | 122 | 62 | 42 | 38 | 56 | 49 | 17 |
| Cheung^110^ | 33 | 2014 | China | East Asian | 2196 | 184 | 2012 | 89 | 83 | 12 | 1007 | 822 | 183 |
| Shaker^30^ | 34 | 2014 | Egypt | African | 120 | 60 | 60 | 44 | 12 | 4 | 56 | 4 | 0 |
| Zhang M^114^ | 35 | 2015 | China | East Asian | 973 | 561 | 412 | 276 | 235 | 50 | 224 | 164 | 24 |
| Du SX^39^ | 36 | 2016 | China | East Asian | 797 | 493 | 304 | 245 | 205 | 43 | 179 | 108 | 17 |
| Mofarrah^45^ | 37 | 2016 | Iran | West Asia | 224 | 152 | 72 | 82 | 35 | 35 | 56 | 13 | 3 |
| Mohammadzadeh^38^ | 38 | 2016 | Iran | West Asia | 200 | 100 | 100 | 75 | 24 | 1 | 65 | 31 | 4 |
| Suo SZ^116^ | 39 | 2016 | China | East Asian | 258 | 128 | 130 | 48 | 66 | 14 | 62 | 56 | 12 |
| Zhang Min^40^ | 40 | 2016 | China | East Asian | 718 | 306 | 412 | 148 | 136 | 22 | 226 | 162 | 24 |

The 70-117 references are listed in Additional file 4

| Table S3 Summary of SNP rs1501299, +276GT characteristics | | | | | | | | | | | | | |
| --- | --- | --- | --- | --- | --- | --- | --- | --- | --- | --- | --- | --- | --- |
| Study | ID | Year | Country | Population | Total | Cases | Controls | CaseGG | CaseGT | CaseTT | ControlGG | ControlGT | ControlTT |
| [Lacquemant Swiss](file:///F:\百度云同步盘\研究僧\卡奴meta分析\数据\表格%20最终%20终删掉outcome.xlsx#RANGE!_ENREF_19)^70^ | 1 | 2004 | Switzerland | European | 285 | 106 | 179 | 57 | 40 | 9 | 96 | 65 | 18 |
| [Lacquemant French](file:///F:\百度云同步盘\研究僧\卡奴meta分析\数据\表格%20最终%20终删掉outcome.xlsx#RANGE!_ENREF_19)^70^ | 2 | 2004 | France | European | 185 | 55 | 130 | 25 | 26 | 4 | 73 | 50 | 7 |
| [Bacci](file:///F:\百度云同步盘\研究僧\卡奴meta分析\数据\表格%20最终%20终删掉outcome.xlsx#RANGE!_ENREF_18)^47^ | 3 | 2004 | Italy | European | 376 | 142 | 234 | 70 | 65 | 7 | 118 | 88 | 28 |
| [Ohashi](file:///F:\百度云同步盘\研究僧\卡奴meta分析\数据\表格%20最终%20终删掉outcome.xlsx#RANGE!_ENREF_59)^71^ | 4 | 2004 | Japan | East Asian | 751 | 383 | 368 | 185 | 164 | 34 | 190 | 149 | 29 |
| [Stenvinkel](file:///F:\百度云同步盘\研究僧\卡奴meta分析\数据\表格%20最终%20终删掉outcome.xlsx#RANGE!_ENREF_60)^41^ | 5 | 2004 | America | European | 204 | 63 | 141 | 38 | 20 | 5 | 62 | 65 | 14 |
| [Filippi](file:///F:\百度云同步盘\研究僧\卡奴meta分析\数据\表格%20最终%20终删掉outcome.xlsx#RANGE!_ENREF_61)^72^ | 6 | 2005 | Italy | European | 1046 | 580 | 466 | 287 | 241 | 52 | 266 | 167 | 33 |
| [Ru Y](file:///F:\百度云同步盘\研究僧\卡奴meta分析\数据\表格%20最终%20终删掉outcome.xlsx#RANGE!_ENREF_62)^73^ | 7 | 2005 | China | East Asian | 267 | 131 | 136 | 79 | 46 | 6 | 64 | 55 | 17 |
| [Qi1](file:///F:\百度云同步盘\研究僧\卡奴meta分析\数据\表格%20最终%20终删掉outcome.xlsx#RANGE!_ENREF_22)^74^ | 8 | 2005 | America | European | 822 | 228 | 594 | 105 | 111 | 12 | 293 | 249 | 52 |
| [Qi2](file:///F:\百度云同步盘\研究僧\卡奴meta分析\数据\表格%20最终%20终删掉outcome.xlsx#RANGE!_ENREF_63)^24^ | 9 | 2006 | America | European | 964 | 280 | 684 | 159 | 104 | 17 | 374 | 258 | 52 |
| [Hegener 1](file:///F:\百度云同步盘\研究僧\卡奴meta分析\数据\表格%20最终%20终删掉outcome.xlsx#RANGE!_ENREF_66)^76^ | 10 | 2006 | America | European | 682 | 341 | 341 | 183 | 134 | 24 | 181 | 143 | 17 |
| [Hegener 2](file:///F:\百度云同步盘\研究僧\卡奴meta分析\数据\表格%20最终%20终删掉outcome.xlsx#RANGE!_ENREF_66)^76^ | 11 | 2006 | America | European | 518 | 259 | 259 | 137 | 98 | 24 | 136 | 104 | 19 |
| Jung^43^ | 12 | 2006 | Korea | East Asian | 156 | 88 | 68 | 38 | 43 | 7 | 31 | 32 | 5 |
| [Gable 1](file:///F:\百度云同步盘\研究僧\卡奴meta分析\数据\表格%20最终%20终删掉outcome.xlsx#RANGE!_ENREF_64)^77^ | 13 | 2007 | UK | European | 2990 | 263 | 2727 | 155 | 96 | 12 | 1511 | 1038 | 178 |
| [Gable 2](file:///F:\百度云同步盘\研究僧\卡奴meta分析\数据\表格%20最终%20终删掉outcome.xlsx#RANGE!_ENREF_64)^77^ | 14 | 2007 | UK | European | 1061 | 504 | 557 | 266 | 216 | 22 | 289 | 225 | 43 |
| Pischon^42^ | 15 | 2007 | America | European | 3093 | 1030 | 2063 | 555 | 393 | 82 | 1039 | 866 | 158 |
| Lu F^78^ | 16 | 2007 | China | East Asian | 266 | 135 | 131 | 74 | 52 | 9 | 47 | 60 | 24 |
| Zhang XL^82^ | 17 | 2009 | China | East Asian | 340 | 205 | 135 | 103 | 85 | 17 | 62 | 59 | 14 |
| Persson^86^ | 18 | 2010 | Sweden | European | 488 | 244 | 244 | 121 | 102 | 21 | 121 | 102 | 21 |
| Caterina^89^ | 19 | 2011 | Italy | European | 3654 | 1833 | 1821 | 926 | 746 | 161 | 906 | 767 | 148 |
| Al-Daghri^90^ | 20 | 2011 | Saudi A. | West Asia | 420 | 123 | 297 | 47 | 57 | 19 | 111 | 142 | 44 |
| Leu^92^ | 21 | 2011 | China | East Asian | 3330 | 80 | 3250 | 39 | 35 | 6 | 1750 | 1261 | 239 |
| Liu F^28^ | 22 | 2011 | China | East Asian | 640 | 302 | 338 | 139 | 128 | 35 | 164 | 142 | 32 |
| Rodriguez^93^ | 23 | 2011 | Spain | European | 674 | 119 | 555 | 69 | 44 | 6 | 287 | 224 | 44 |
| Boumaiza^100^ | 24 | 2011 | Tunisia | African | 316 | 212 | 104 | 105 | 84 | 23 | 45 | 41 | 18 |
| Esteghamati^48^ | 25 | 2012 | Iran | West Asia | 241 | 114 | 127 | 76 | 30 | 8 | 63 | 47 | 17 |
| Gui^102^ | 26 | 2012 | China | East Asian | 841 | 410 | 431 | 172 | 185 | 53 | 239 | 154 | 38 |
| Katakami^23^ | 27 | 2012 | Japan | East Asian | 2637 | 213 | 2424 | 129 | 71 | 13 | 1230 | 975 | 219 |
| Oliveira^44^ | 28 | 2012 | Brazil | European | 603 | 450 | 153 | 209 | 197 | 44 | 63 | 68 | 22 |
| Shi KL^103^ | 29 | 2012 | China | East Asian | 688 | 396 | 292 | 208 | 155 | 33 | 120 | 127 | 45 |
| Zhang HF^104^ | 30 | 2012 | China | East Asian | 512 | 394 | 118 | 18 | 167 | 209 | 13 | 51 | 54 |
| Nannan^105^ | 31 | 2012 | China | East Asian | 680 | 213 | 467 | 115 | 84 | 14 | 237 | 191 | 39 |
| Antonopoulos^106^ | 32 | 2013 | Greece | European | 594 | 462 | 132 | 220 | 212 | 30 | 66 | 50 | 16 |
| Rizk^107^ | 33 | 2013 | Qatar | West Asia | 263 | 142 | 121 | 58 | 64 | 20 | 46 | 59 | 16 |
| Wang CH^108^ | 34 | 2013 | China | East Asian | 217 | 101 | 116 | 52 | 38 | 11 | 49 | 47 | 20 |
| Wu/276^109^ | 35 | 2013 | China | East Asian | 388 | 188 | 200 | 67 | 108 | 13 | 92 | 90 | 18 |
| Cheung^110^ | 36 | 2014 | China | East Asian | 2192 | 182 | 2010 | 88 | 75 | 19 | 1103 | 759 | 148 |
| Foucan 2^49^ | 37 | 2014 | France | African | 194 | 52 | 142 | 19 | 25 | 8 | 53 | 68 | 21 |
| Li Yang^111^ | 38 | 2014 | China | East Asian | 599 | 234 | 365 | 115 | 96 | 23 | 154 | 169 | 42 |
| Torres^113^ | 39 | 2015 | Portugal | European | 100 | 43 | 57 | 22 | 19 | 2 | 27 | 24 | 6 |
| Zhang M^114^ | 40 | 2015 | China | East Asian | 973 | 561 | 412 | 309 | 209 | 43 | 214 | 170 | 28 |
| Liu Yun^115^ | 41 | 2015 | China | East Asian | 400 | 200 | 200 | 126 | 57 | 17 | 145 | 46 | 9 |
| Mohammadzadeh^38^ | 42 | 2016 | Iran | West Asia | 200 | 100 | 100 | 38 | 55 | 7 | 56 | 42 | 2 |
| Zhang Min^40^ | 43 | 2016 | China | East Asian | 718 | 306 | 412 | 175 | 109 | 22 | 214 | 170 | 28 |
| Li SS^117^ | 44 | 2017 | China | East Asian | 803 | 385 | 418 | 193 | 145 | 47 | 220 | 158 | 40 |

The 70-117 references are listed in Additional file 4
