# Supplementary material for: Associations between three common single nucleotide polymorphisms (rs266729, rs2241766, and rs1501299) of ADIPOQ and cardiovascular disease: a meta-analysis
Source: Lipids Health Dis. 2018 May 28;17:126. doi: 10.1186/s12944-018-0767-8 (PMC5972450; doi:10.1186/s12944-018-0767-8)
Supplement: Supplementary file 2 — Meta-regression results of the association between the SNPs and CVD risk. (DOCX 59 kb) [file 12944_2018_767_MOESM2_ESM.docx]

| Table S4 Meta-regression results of the association between the SNPs and CVD risk. | | | | | | | | |
| --- | --- | --- | --- | --- | --- | --- | --- | --- |
| SNPs | Genetic model | Covariates | Coefficient | Std. Err. | *P* | 95% *CI* | *τ^2^*value (%) | *I^2^* (%) |
| rs266729 | Allelic model | **Year** | -0.001 | 0.012 | 0.954 | -0.026~0.025 | 3.279 | 67.03 |
|  |  | **Population** |  |  |  |  | 2.871 | 63.01 |
|  |  | European | -0.117 | 0.083 | 0.170 | -0.053~0.288 |  |  |
|  |  | East Asian | referent | referent | referent | referent |  |  |
|  |  | **Genotyping method** |  |  |  |  | 2.980 | 64.48 |
|  |  | PCR-RFLP | 0.131 | 0.110 | 0.246 | -0.356~0.095 |  |  |
|  |  | TaqMan | -0.007 | 0.099 | 0.942 | -0.197~0.212 |  |  |
|  |  | Other methods | referent | referent | referent | referent |  |  |
|  |  | **Sample size** |  |  |  |  | 3.243 | 66.82 |
|  |  | ≥ 1000 | 0.043 | 0.090 | 0.635 | -0.228~0.141 |  |  |
|  |  | < 1000 | referent | referent | referent | referent |  |  |
|  |  | **Quality score** |  |  |  |  | 2.796 | 63.44 |
|  |  | < 10 | 0.128 | 0.082 | 0.129 | -0.296~0.040 |  |  |
|  |  | ≥ 10 | referent | referent | referent | referent |  |  |
|  | Dominant model | **Year** | -0.004 | 0.015 | 0.773 | -0.034~0.026 | 4.463 | 65.69 |
|  |  | **Population** |  |  |  |  | 4.118 | 62.62 |
|  |  | European | -0.104 | 0.101 | 0.313 | -0.311~0.103 |  |  |
|  |  | East Asian | referent | referent | referent | referent |  |  |
|  |  | **Genotyping method** |  |  |  |  | 3.952 | 62.76 |
|  |  | PCR-RFLP | 0.155 | 0.130 | 0.243 | -0.112~0.423 |  |  |
|  |  | TaqMan | -0.029 | 0.117 | 0.804 | -0.271~0.212 |  |  |
|  |  | Other methods | referent | referent | referent | referent |  |  |
|  |  | **Sample size** |  |  |  |  | 3.243 | 66.82 |
|  |  | ≥ 1000 | 0.047 | 0.107 | 0.661 | -0.172~0.267 |  |  |
|  |  | < 1000 | referent | referent | referent | referent |  |  |
|  |  | **Quality score** |  |  |  |  | 3.995 | 63.22 |
|  |  | < 10 | 0.134 | 0.099 | 0.187 | -0.069~0.336 |  |  |
|  |  | ≥ 10 | referent | referent | referent | referent |  |  |
|  | Recessive model | **Year** | 0.012 | 0.028 | 0.680 | -0.046~0.070 | 8.601 | 50.06 |
|  |  | **Population** |  |  |  |  | 6.716 | 45.79 |
|  |  | European | -0.269 | 0.178 | 0.143 | -0.635~0.097 |  |  |
|  |  | East Asian | referent | referent | referent | referent |  |  |
|  |  | **Genotyping method** |  |  |  |  | 8.731 | 49.95 |
|  |  | PCR-RFLP | 0.251 | 0.247 | 0.319 | -0.257~0.758 |  |  |
|  |  | TaqMan | -0.045 | 0.222 | 0.842 | -0.411~0.500 |  |  |
|  |  | Other methods | referent | referent | referent | referent |  |  |
|  |  | **Sample size** |  |  |  |  | 8.911 | 50.33 |
|  |  | ≥ 1000 | 0.060 | 0.193 | 0.757 | -0.335~0.455 |  |  |
|  |  | < 1000 | referent | referent | referent | referent |  |  |
|  |  | **Quality score** |  |  |  |  | 4.413 | 42.93 |
|  |  | < 10 | 0.304 | 0.173 | 0.091 | -0.051~0.659 |  |  |
|  |  | ≥ 10 | referent | referent | referent | referent |  |  |
|  | Heterozygote model | **Year** | -0.006 | 0.014 | 0.662 | -0.036~0.023 | 3.771 | 60.99 |
|  |  | **Population** |  |  |  |  | 3.627 | 58.99 |
|  |  | European | -0.065 | 0.100 | 0.520 | -0.269~0.139 |  |  |
|  |  | East Asian | referent | referent | referent | referent |  |  |
|  |  | **Genotyping method** |  |  |  |  | 3.352 | 58.42 |
|  |  | PCR-RFLP | 0.136 | 0.127 | 0.292 | -0.124~0.397 |  |  |
|  |  | TaqMan | -0.044 | 0.114 | 0.703 | -0.279~0.191 |  |  |
|  |  | Other methods | referent | referent | referent | referent |  |  |
|  |  | **Sample size** |  |  |  |  | 3.758 | 61.08 |
|  |  | ≥ 1000 | 0.044 | 0.103 | 0.673 | -0.168~0.256 |  |  |
|  |  | < 1000 | referent | referent | referent | referent |  |  |
|  |  | **Quality score** |  |  |  |  | 3.554 | 59.69 |
|  |  | < 10 | 0.102 | 0.977 | 0.304 | -0.098~0.303 |  |  |
|  |  | ≥ 10 | referent | referent | referent | referent |  |  |
|  | Homozygote model | **Year** | 0.011 | 0.031 | 0.727 | -0.052~0.074 | 12.380 | 54.96 |
|  |  | **Population** |  |  |  |  | 9.628 | 49.86 |
|  |  | European | -0.312 | 0.194 | 0.118 | -0.710~0.085 |  |  |
|  |  | East Asian | referent | referent | referent | referent |  |  |
|  |  | **Genotyping method** |  |  |  |  | 1.136 | 53.26 |
|  |  | PCR-RFLP | 0.329 | 0.265 | 0.225 | -0.215~0.873 |  |  |
|  |  | TaqMan | 0.043 | 0.238 | 0.859 | -0.446~0.531 |  |  |
|  |  | Other methods | referent | referent | referent | referent |  |  |
|  |  | **Sample size** |  |  |  |  | 1.251 | 54.99 |
|  |  | ≥ 1000 | 0.093 | 0.211 | 0.663 | -0.340~0.526 |  |  |
|  |  | < 1000 | referent | referent | referent | referent |  |  |
|  |  | **Quality score** |  |  |  |  | 7.942 | 48.14 |
|  |  | < 10 | 0.327 | 0.191 | 0.099 | -0.065~0.719 |  |  |
|  |  | ≥ 10 | referent | referent | referent | referent |  |  |
| rs2241766 | Allelic model | **Year** | 0.016 | 0.018 | 0.368 | -0.020~0.052 | 76.160 | 71.32 |
|  |  | **Population** |  |  |  |  | 6.716 | 70.29 |
|  |  | East Asian | -0.409 | 0.306 | 0.190 | -0.212~1.031 |  |  |
|  |  | European | -0.503 | 0.314 | 0.119 | -0.135~1.141 |  |  |
|  |  | West Asian | -0.148 | 0.344 | 0.670 | -0.549~0.845 |  |  |
|  |  | African | referent | referent | referent | referent |  |  |
|  |  | **Genotyping method** |  |  |  |  | 8.240 | 73.25 |
|  |  | PCR-RFLP | -0.032 | 0.139 | 0.820 | -0.250~0.313 |  |  |
|  |  | TaqMan | -0.148 | 0.181 | 0.417 | -0.218~0.514 |  |  |
|  |  | Other methods | referent | referent | referent | referent |  |  |
|  |  | **Sample size** |  |  |  |  | 5.202 | 63.92 |
|  |  | < 1000 | 0.333 | 0.136 | **0.019** | -0.609~0.057 |  |  |
|  |  | ≥ 1000 | referent | referent | referent | referent |  |  |
|  |  | **Quality score** |  |  |  |  | 6.393 | 70.59 |
|  |  | ≥ 10 | -0.253 | 0.116 | **0.035** | 0.019~0.487 |  |  |
|  |  | < 10 | referent | referent | referent | referent |  |  |
|  | Dominant model | **Year** | 0.015 | 0.019 | 0.442 | -0.024~0.053 | 7.859 | 64.43 |
|  |  | **Population** |  |  |  |  | 8.008 | 65.42 |
|  |  | East Asian | -0.436 | 0.328 | 0.193 | -1.102~0.230 |  |  |
|  |  | European | -0.518 | 0.336 | 0.133 | -1.200~0.165 |  |  |
|  |  | West Asian | -0.310 | 0.373 | 0.410 | -1.066~0.445 |  |  |
|  |  | African | referent | referent | referent | referent |  |  |
|  |  | **Genotyping method** |  |  |  |  | 8.804 | 66.95 |
|  |  | PCR-RFLP | 0.001 | 0.149 | 0.995 | -0.300~0.302 |  |  |
|  |  | TaqMan | -0.104 | 0.190 | 0.588 | -0.489~0.281 |  |  |
|  |  | Other methods | referent | referent | referent | referent |  |  |
|  |  | **Sample size** |  |  |  |  | 5.577 | 58.47 |
|  |  | < 1000 | 0.320 | 0.144 | **0.032** | 0.030~0.611 |  |  |
|  |  | ≥ 1000 | referent | referent | referent | referent |  |  |
|  |  | **Quality score** |  |  |  |  | 6.522 | 63.29 |
|  |  | ≥ 10 | -0.270 | 0.122 | **0.033** | -0.517~0.023 |  |  |
|  |  | < 10 | referent | referent | referent | referent |  |  |
|  | Recessive model | **Year** | 0.043 | 0.029 | 0.155 | -0.017~0.103 | 13.390 | 47.16 |
|  |  | **Population** |  |  |  |  | 7.100 | 32.36 |
|  |  | East Asian | -0.034 | 0.584 | 0.953 | -1.222~1.153 |  |  |
|  |  | European | -0.543 | 0.603 | 0.375 | -1.769~0.684 |  |  |
|  |  | West Asian | 0.697 | 0.626 | 0.273 | -0.576~1.970 |  |  |
|  |  | African | referent | referent | referent | referent |  |  |
|  |  | **Genotyping method** |  |  |  |  | 14.770 | 49.66 |
|  |  | Other methods | 0.489 | 0.290 | 0.100 | -0.099~1.078 |  |  |
|  |  | PCR-RFLP | 0.413 | 0.277 | 0.145 | -0.149~0.974 |  |  |
|  |  | TaqMan | referent | referent | referent | referent |  |  |
|  |  | **Sample size** |  |  |  |  | 0.000 | 18.67 |
|  |  | < 1000 | 0.797 | 0.163 | **<0.001** | 0.466~1.129 |  |  |
|  |  | ≥ 1000 | referent | referent | referent | referent |  |  |
|  |  | **Quality score** |  |  |  |  | 13.240 | 47.47 |
|  |  | ≥ 10 | 0.439 | 0.193 | **0.029** | 0.049~0.830 |  |  |
|  |  | < 10 | referent | referent | referent | referent |  |  |
|  | Heterozygote model | **Year** | 0.009 | 0.016 | 0.594 | -0.025~0.042 | 3.961 | 52.96 |
|  |  | **Population** |  |  |  |  | 4.443 | 54.67 |
|  |  | East Asian | -0.414 | 0.309 | 0.189 | -1.041~0.212 |  |  |
|  |  | European | -0.456 | 0.315 | 0.156 | -0.109~0.183 |  |  |
|  |  | West Asian | -0.483 | 0.352 | 0.178 | -1.197~0.230 |  |  |
|  |  | African | referent | referent | referent | referent |  |  |
|  |  | **Genotyping method** |  |  |  |  | 4.837 | 55.59 |
|  |  | PCR-RFLP | 0.024 | 0.130 | 0.854 | -0.239~0.288 |  |  |
|  |  | TaqMan | -0.245 | 0.162 | 0.881 | -0.354~0.305 |  |  |
|  |  | Other methods | referent | referent | referent | referent |  |  |
|  |  | **Sample size** |  |  |  |  | 2.707 | 48.37 |
|  |  | < 1000 | -0.218 | 0.118 | 0.073 | -0.022~0.458 |  |  |
|  |  | ≥ 1000 | referent | referent | referent | referent |  |  |
|  |  | **Quality score** |  |  |  |  | 3.648 | 51.60 |
|  |  | ≥ 10 | -0.204 | 0.107 | 0.063 | -0.419~0.012 |  |  |
|  |  | < 10 | referent | referent | referent | referent |  |  |
|  | Homozygote model | **Year** | 0.047 | 0.033 | 0.158 | -0.019~0.114 | 19.490 | 55.23 |
|  |  | **Population** |  |  |  |  | 12.450 | 47.65 |
|  |  | East Asian | -0.057 | 0.626 | 0.928 | -1.329~1.215 |  |  |
|  |  | European | -0.622 | 0.646 | 0.343 | -1.935~0.691 |  |  |
|  |  | West Asian | 0.623 | 0.676 | 0.363 | -0.750~2.000 |  |  |
|  |  | African | referent | referent | referent | referent |  |  |
|  |  | **Genotyping method** |  |  |  |  | 21.370 | 58.12 |
|  |  | Other methods | 0.517 | 0.324 | 0.119 | -0.140~1.174 |  |  |
|  |  | PCR-RFLP | 0.464 | 0.309 | 0.142 | -0.163~1.092 |  |  |
|  |  | TaqMan | referent | referent | referent | referent |  |  |
|  |  | **Sample size** |  |  |  |  | 3.640 | 30.26 |
|  |  | < 1000 | 0.876 | 0.202 | **<0.001** | 0.467~1.285 |  |  |
|  |  | ≥ 1000 | referent | referent | referent | referent |  |  |
|  |  | **Quality score** |  |  |  |  | 17.820 | 55.05 |
|  |  | < 10 | 0.537 | 0.210 | **0.015** | 0.112~0.963 |  |  |
|  |  | ≥ 10 | referent | referent | referent | referent |  |  |
| rs1501299 | Allelic model | **Year** | 0.009 | 0.010 | 0.360 | -0.011~0.029 | 3.745 | 65.24 |
|  |  | **Population** |  |  |  |  | 4.029 | 66.82 |
|  |  | East Asian | 0.118 | 0.219 | 0.595 | -0.561~0.325 |  |  |
|  |  | European | 0.092 | 0.220 | 0.677 | -0.537~0.352 |  |  |
|  |  | West Asian | 0.119 | 0.256 | 0.644 | -0.637~0.399 |  |  |
|  |  | African | referent | referent | referent | referent |  |  |
|  |  | **Genotyping method** |  |  |  |  | 3.921 | 66.16 |
|  |  | Other methods | -0.046 | 0.095 | 0.628 | -0.146~0.238 |  |  |
|  |  | PCR-RFLP | -0.002 | 0.100 | 0.981 | -0.199~0.204 |  |  |
|  |  | TaqMan | referent | referent | referent | referent |  |  |
|  |  | **Sample size** |  |  |  |  | 3.825 | 65.50 |
|  |  | ≥ 1000 | 0.043 | 0.092 | 0.648 | -0.229~0.144 |  |  |
|  |  | < 1000 | referent | referent | referent | referent |  |  |
|  |  | **Quality score** |  |  |  |  | 3.821 | 65.56 |
|  |  | < 10 | 0.002 | 0.077 | 0.981 | -0.158~0.154 |  |  |
|  |  | ≥ 10 | referent | referent | referent | referent |  |  |
|  | Dominant model | **Year** | 0.007 | 0.012 | 0.571 | -0.018~0.032 | 5.101 | 61.43 |
|  |  | **Population** |  |  |  |  | 5.431 | 63.12 |
|  |  | East Asian | 0.113 | 0.286 | 0.695 | -0.465~0.691 |  |  |
|  |  | European | 0.103 | 0.287 | 0.720 | -0.476~0.683 |  |  |
|  |  | West Asian | 0.087 | 0.333 | 0.795 | -0.586~0.759 |  |  |
|  |  | African | referent | referent | referent | referent |  |  |
|  |  | **Genotyping method** |  |  |  |  | 5.261 | 62.18 |
|  |  | Other methods | -0.050 | 0.117 | 0.668 | -0.286~0.185 |  |  |
|  |  | PCR-RFLP | 0.010 | 0.124 | 0.937 | -0.240~0.260 |  |  |
|  |  | TaqMan | referent | referent | referent | referent |  |  |
|  |  | **Sample size** |  |  |  |  | 5.122 | 61.47 |
|  |  | ≥ 1000 | 0.315 | 0.112 | 0.780 | -0.195~0.258 |  |  |
|  |  | < 1000 | referent | referent | referent | referent |  |  |
|  |  | **Quality score** |  |  |  |  | 5.084 | 61.42 |
|  |  | < 10 | 0.015 | 0.096 | 0.874 | -0.178~0.208 |  |  |
|  |  | ≥ 10 | referent | referent | referent | referent |  |  |
|  | Recessive model | **Year** | 0.024 | 0.017 | 0.164 | -0.010~0.058 | 6.435 | 41.25 |
|  |  | **Population** |  |  |  |  | 7.123 | 44.61 |
|  |  | East Asian | 0.255 | 0.352 | 0.474 | -0.457~0.967 |  |  |
|  |  | European | 0.126 | 0.354 | 0.723 | -0.590~0.842 |  |  |
|  |  | West Asian | 0.293 | 0.423 | 0.493 | -0.563~1.149 |  |  |
|  |  | African | referent | referent | referent | referent |  |  |
|  |  | **Genotyping method** |  |  |  |  | 7.150 | 44.06 |
|  |  | Other methods | -0.103 | 0.159 | 0.521 | -0.423~0.218 |  |  |
|  |  | PCR-RFLP | -0.073 | 0.166 | 0.662 | -0.408~0.262 |  |  |
|  |  | TaqMan | referent | referent | referent | referent |  |  |
|  |  | **Sample size** |  |  |  |  | 6.861 | 42.35 |
|  |  | ≥ 1000 | 0.084 | 0.148 | 0.572 | -0.214~0.383 |  |  |
|  |  | < 1000 | referent | referent | referent | referent |  |  |
|  |  | **Quality score** |  |  |  |  | 6.918 | 43.06 |
|  |  | < 10 | -0.030 | 0.129 | 0.819 | -0.290~0.231 |  |  |
|  |  | ≥ 10 | referent | referent | referent | referent |  |  |
|  | Heterozygote model | **Year** | 0.002 | 0.011 | 0.890 | -0.021~0.024 | 3.206 | 50.36 |
|  |  | **Population** |  |  |  |  | 3.435 | 52.67 |
|  |  | East Asian | 0.055 | 0.273 | 0.840 | -0.496~0.606 |  |  |
|  |  | European | 0.067 | 0.273 | 0.808 | -0.485~0.618 |  |  |
|  |  | West Asian | 0.017 | 0.316 | 0.957 | -0.622~0.657 |  |  |
|  |  | African | referent | referent | referent | referent |  |  |
|  |  | **Genotyping method** |  |  |  |  | 3.323 | 51.22 |
|  |  | Other methods | -0.041 | 0.103 | 0.692 | -0.249~0.167 |  |  |
|  |  | PCR-RFLP | 0.012 | 0.110 | 0.912 | -0.211~0.235 |  |  |
|  |  | TaqMan | referent | referent | referent | referent |  |  |
|  |  | **Sample size** |  |  |  |  | 3.239 | 50.22 |
|  |  | ≥ 1000 | 0.007 | 0.097 | 0.942 | -0.188~0.202 |  |  |
|  |  | < 1000 | referent | referent | referent | referent |  |  |
|  |  | **Quality score** |  |  |  |  | 3.170 | 50.07 |
|  |  | < 10 | 0.025 | 0.085 | 0.775 | -0.147~0.197 |  |  |
|  |  | ≥ 10 | referent | referent | referent | referent |  |  |
|  | Homozygote model | **Year** | 0.027 | 0.021 | 0.205 | -0.015~0.069 | 13.020 | 55.46 |
|  |  | **Population** |  |  |  |  | 14.160 | 57.74 |
|  |  | East Asian | 0.274 | 0.432 | 0.529 | -0.599~1.147 |  |  |
|  |  | European | 0.132 | 0.434 | 0.762 | -0.744~1.009 |  |  |
|  |  | West Asian | 0.273 | 0.516 | 0.600 | -0.770~1.317 |  |  |
|  |  | African | referent | referent | referent | referent |  |  |
|  |  | **Genotyping method** |  |  |  |  | 13.990 | 57.06 |
|  |  | Other methods | -0.120 | 0.196 | 0.544 | -0.517~0.276 |  |  |
|  |  | PCR-RFLP | -0.049 | 0.207 | 0.815 | -0.467~0.369 |  |  |
|  |  | TaqMan | referent | referent | referent | referent |  |  |
|  |  | **Sample size** |  |  |  |  | 13.590 | 56.05 |
|  |  | ≥ 1000 | 0.086 | 0.188 | 0.650 | -0.294~0.466 |  |  |
|  |  | < 1000 | referent | referent | referent | referent |  |  |
|  |  | **Quality score** |  |  |  |  | 13.620 | 56.36 |
|  |  | < 10 | -0.014 | 0.161 | 0.931 | -0.338~0.310 |  |  |
|  |  | ≥ 10 | referent | referent | referent | referent |  |  |
| Bold values indicated that these covariates were the sources of heterogeneity (*P* value < 0.05) | | | | | | | | |
| Std. Err.: standard error; CI: confidence interval | | | | | | | | |
