# Supplementary material for: Associations between three common single nucleotide polymorphisms (rs266729, rs2241766, and rs1501299) of ADIPOQ and cardiovascular disease: a meta-analysis
Source: Lipids Health Dis. 2018 May 28;17:126. doi: 10.1186/s12944-018-0767-8 (PMC5972450; doi:10.1186/s12944-018-0767-8)
Supplement: Supplementary file 3 — Funnel plots of three SNPs for publication bias. (DOCX 1250 kb) [file 12944_2018_767_MOESM3_ESM.docx]

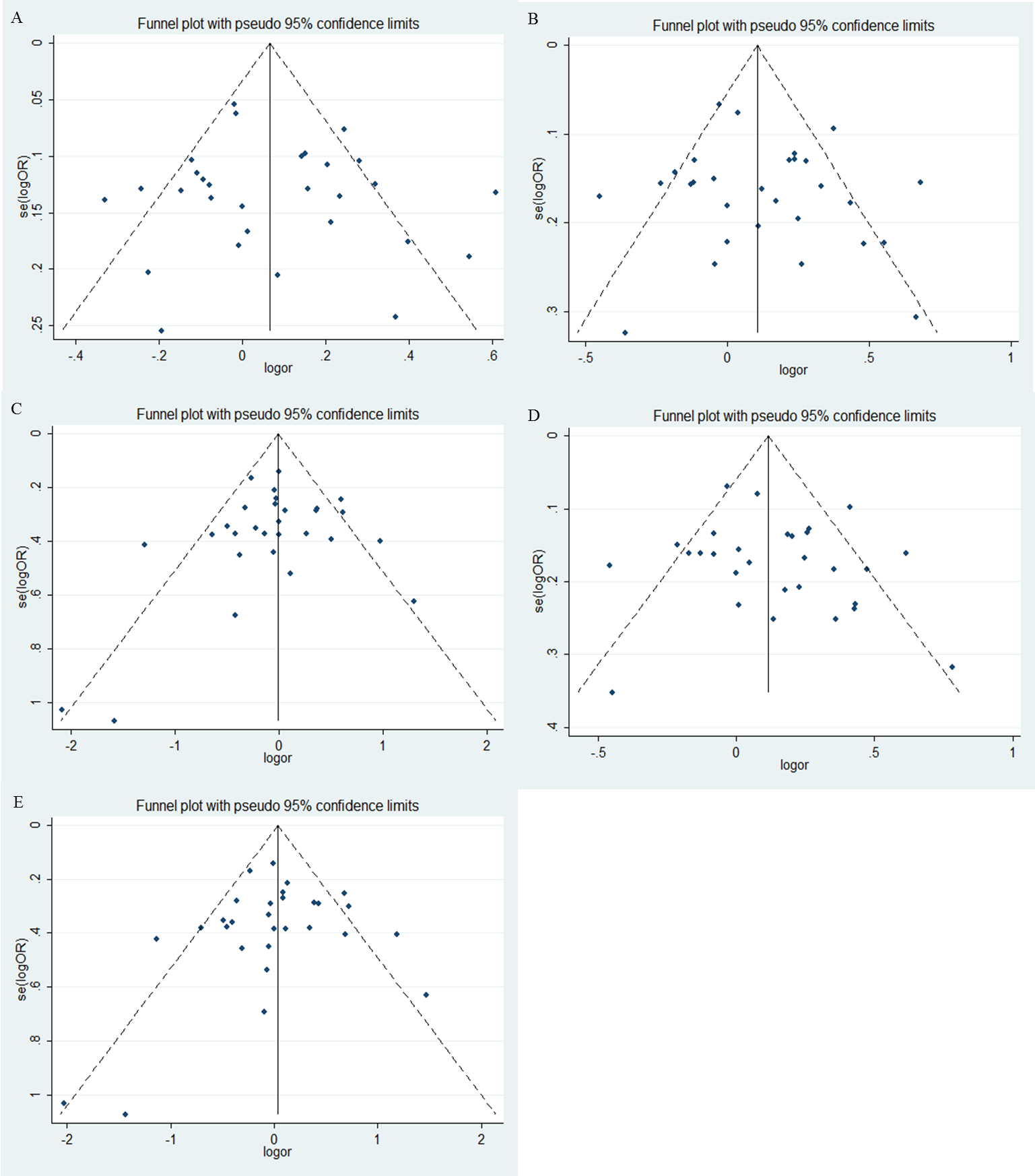


Figure S1: Funnel plots of rs266729 for publication bias. (A) allelic model; (B) dominant model; (C) recessive model; (D) heterozygote model; (E) homozygote model.


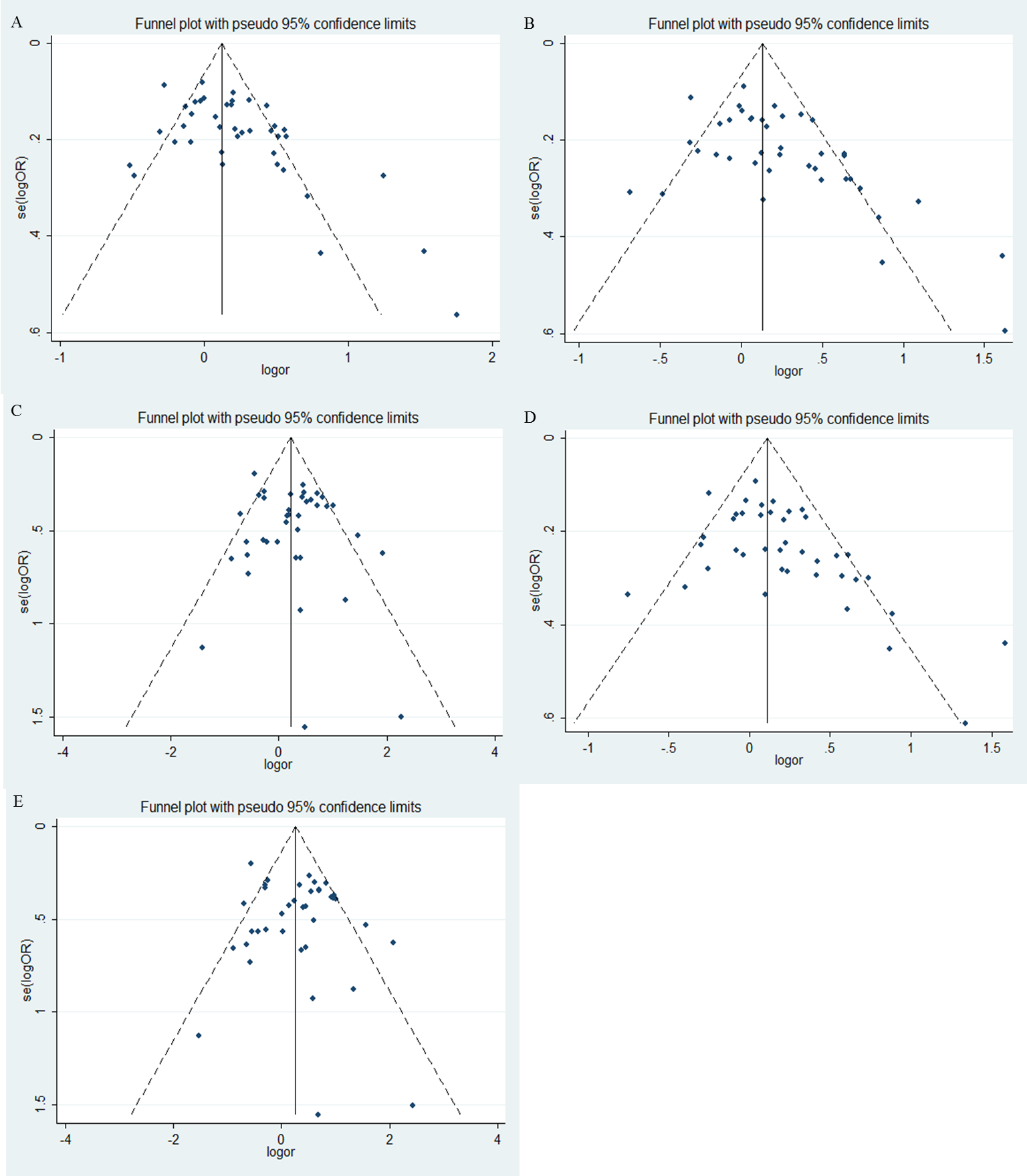


Figure S2: Funnel plots of rs2241766 for publication bias. (A) allelic model; (B) dominant model; (C) recessive model; (D) heterozygote model; (E) homozygote model.


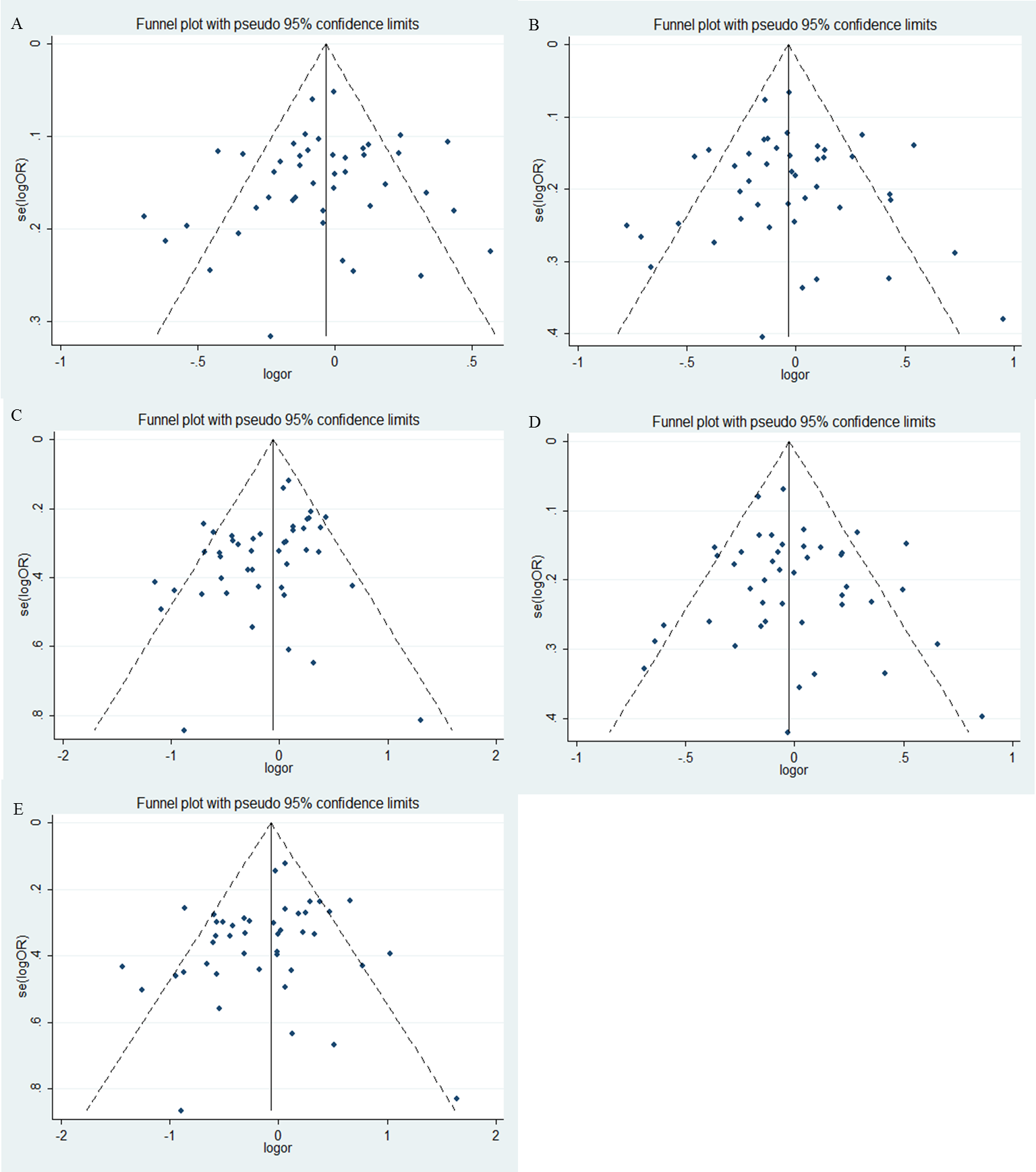


Figure S3: Funnel plots of rs1501299 for publication bias. (A) allelic model; (B) dominant model; (C) recessive model; (D) heterozygote model; (E) homozygote model.
