# Supplementary material for: Associations between three common single nucleotide polymorphisms (rs266729, rs2241766, and rs1501299) of ADIPOQ and cardiovascular disease: a meta-analysis
Source: Lipids Health Dis. 2018 May 28;17:126. doi: 10.1186/s12944-018-0767-8 (PMC5972450; doi:10.1186/s12944-018-0767-8)
Supplement: Supplementary file 4 — Additional references. (DOCX 22 kb) [file 12944_2018_767_MOESM4_ESM.docx]

Additional references

Note:

Our meta-analysis includes the references (70-117) that are not listed in main text. The references (70-117) are cited in the Table 1 and in Additional file 1 (Tables S1-3). We list these references here for readers to retrieve them conveniently.

70. Lacquemant C, Froguel P, Lobbens S, Izzo P, Dina C, Ruiz J. The adiponectin gene SNP+45 is associated with coronary artery disease in Type 2 (non-insulin-dependent) diabetes mellitus. Diabet Med. 2004; 21: 776-81.

71. Ohashi K, Ouchi N, Kihara S, Funahashi T, Nakamura T, Sumitsuji S, Kawamoto T, Matsumoto S, Nagaretani H, Kumada M, Okamoto Y, Nishizawa H, Kishida K, Maeda N, Hiraoka H, Iwashima Y, Ishikawa K, Ohishi M, Katsuya T, Rakugi H, Ogihara T, Matsuzawa Y. Adiponectin I164T mutation is associated with the metabolic syndrome and coronary artery disease. J Am Coll Cardiol. 2004; 43: 1195-200.

72. Filippi E, Sentinelli F, Romeo S, Arca M, Berni A, Tiberti C, Verrienti A, Fanelli M, Fallarino M, Sorropago G, Baroni MG. The adiponectin gene SNP+276G>T associates with early-onset coronary artery disease and with lower levels of adiponectin in younger coronary artery disease patients (age <or=50 years). J Mol Med (Berl). 2005; 83: 711-9.

73. Ru Y, Ma M, Ma T, Wang C, Wang Y, Zhang Q, Chen M, Yang M. Relations of adiponectin gene SNP276 with coronary heart disease and insulin sensitivity. Chinese Circulation J. 2005; 20: 14-7.

74. Qi L, Li T, Rimm E, Zhang C, Rifai N, Hunter D, Doria A, Hu FB. The +276 polymorphism of the APM1 gene, plasma adiponectin concentration, and cardiovascular risk in diabetic men. Diabetes. 2005; 54: 1607-10.

75. Wang J, Li S. Adiponectin gene polymorphism in Han people of Jilin region and its association with coronary heart disease. Chin J Lab Diag. 2006; 10: 1272-4.

76. Hegener HH, Lee IM, Cook NR, Ridker PM, Zee RY. Association of adiponectin gene variations with risk of incident myocardial infarction and ischemic stroke: a nested case-control study. Clin Chem. 2006; 52: 2021-7.

77. Gable DR, Matin J, Whittall R, Cakmak H, Li KW, Cooper J, Miller GJ, Humphries SE. Common adiponectin gene variants show different effects on risk of cardiovascular disease and type 2 diabetes in European subjects. Ann Hum Genet. 2007; 71: 453-66.

78. Lu F, Wang L, Yang Z, Zhu T, Wang Z, Zhu H, Tang N, Cao K, Huang J, Du M. Correlation between single nucleotide polymorphism (SNP+276G/T) of adiponectin gene and coronary artery disease in non-diabetic population. J Nanjing Med Univ. 2007; 27: 736-9.

79. Hoefle G, Muendlein A, Saely CH, Risch L, Rein P, Koch L, Schmid F, Aczel S, Marte T, Langer P, Drexel H. The -11377 C>G promoter variant of the adiponectin gene, prevalence of coronary atherosclerosis, and incidence of vascular events in men. Thromb Haemost. 2007; 97: 451-7.

80. Yamada Y, Kato K, Oguri M, Yoshida T, Yokoi K, Watanabe S, Metoki N, Yoshida H, Satoh K, Ichihara S, Aoyagi Y, Yasunaga A, Park H, Tanaka M, Nozawa Y. Association of genetic variants with atherothrombotic cerebral infarction in Japanese individuals with metabolic syndrome. Int J Mol Med. 2008; 21: 801-8.

81. Oguri M, Kato K, Yokoi K, Itoh T, Yoshida T, Watanabe S, Metoki N, Yoshida H, Satoh K, Aoyagi Y, Nishigaki Y, Tanaka M, Nozawa Y, Yamada Y. Association of genetic variants with myocardial infarction in Japanese individuals with metabolic syndrome. Atherosclerosis. 2009; 206: 486-93.

82. Zhang X, Peng W, Lu L, Wu L, Gu G, Wang L, Yan X, Chen Q, Shen W. Correlation of adiponectin gene polymorphisms with coronary artery disease and plaque progression. J Shanghai Jiaotong Univ (Med Sci). 2009; 29: 435-9.

83. Zhong C, Zhen D, Qi Q, Genshan M. A lack of association between adiponectin polymorphisms and coronary artery disease in a Chinese population. Genet Mol Biol. 2010; 33: 428-33.

84. Foucan L, Ezourhi N, Maimaitiming S, Hedreville S, Inamo J, Atallah A, Bangou-Bredent J, Aubert R, Chout R, Fumeron F, Donnet JP, Marre M. Adiponectin multimers and ADIPOQ T45G in coronary artery disease in Caribbean type 2 diabetic subjects of African descent. Obesity (Silver Spring). 2010; 18: 1466-8.

85. Xu L, Ling W. Correlation of adiponectin gene SNP +45T/G polymorphism with coronary heart disease. Chinese J Pathophysiology. 2010; 26: 1064-8.

86. Persson J, Lindberg K, Gustafsson TP, Eriksson P, Paulsson-Berne G, Lundman P. Low plasma adiponectin concentration is associated with myocardial infarction in young individuals. J Intern Med. 2010; 268: 194-205.

87. Chen X, Cheng J, Zhang R, Liu J, Li X, Tong Y, Geng Y. Study on the relationship between polymorphism of adiponectin gene and risk of ischemic stroke among Han population in the Northern parts of China. Chinese Journal of Epidemiology. 2010; 31: 129-32.

88. Luo S, Lei H, Liu Q, Ma K, Xiang L, Cao J. Study on the relationship between adiponectin +45 nucleotide T/G polymorphism and coronary heart disease. Chongqing Med J. 2010; 39:1517-9.

89. De Caterina R, Talmud PJ, Merlini PA, Foco L, Pastorino R, Altshuler D, Mauri F, Peyvandi F, Lina D, Kathiresan S, Bernardinelli L, Ardissino D. Strong association of the APOA5-1131T>C gene variant and early-onset acute myocardial infarction. Atherosclerosis. 2011; 214: 397-403.

90. Al-Daghri NM, Al-Attas OS, Alokail MS, Alkharfy KM, Hussain T. Adiponectin gene variants and the risk of coronary artery disease in patients with type 2 diabetes. Mol Biol Rep. 2011; 38: 3703-8.

91. Prior SL, Jones DA, Gill GV, Bain SC, Stephens JW. Association of the adiponectin rs266729 C>G variant and coronary heart disease in the low risk 'Golden Years' type 1 diabetes cohort. Diabetes Res Clin Pract. 2011; 91: e71-4.

92. Leu HB, Chung CM, Chuang SY, Bai CH, Chen JR, Chen JW, Pan WH. Genetic variants of connexin37 are associated with carotid intima-medial thickness and future onset of ischemic stroke. Atherosclerosis. 2011; 214: 101-6.

93. Rodriguez-Rodriguez L, Garcia-Bermudez M, Gonzalez-Juanatey C, Vazquez-Rodriguez TR, Miranda-Filloy JA, Fernandez-Gutierrez B, Llorca J, Martin J, Gonzalez-Gay MA. Lack of association between ADIPOQ rs266729 and ADIPOQ rs1501299 polymorphisms and cardiovascular disease in rheumatoid arthritis patients. Tissue Antigens. 2011; 77: 74-8.

94. Chen F, Wu H, Wang J, Yang R. Relationship between adiponectin level and its gene polymorphism with coronary heart disease. J Clin Cardiol (China). 2011; 27: 284-7.

95. Maimaitiyiming D, Aizezi R, Yumusi K. Adiponectin gene polymorphism in Uygur people of Urumqi region and its association with coronary heart disease. J Clinical Rational Drug Use. 2011; 4: 36-7.

96. Hu H, Jiang X, Shen F, Shen J, Xu H. Adiponectin gene polymorphism in Han People of Jiaxing region and its association with coronary heart disease. J Jiaxing Univ. 2011; 23: 52-5.

97. Zhang Y, Yang Y, Sun Y. Association of AdipoQ SNP and coronary heart disease in the elderly. Shandong Med J. 2011; 51: 107-8.

98. Zhou N, Zhuo J, Wang J, Li X, Ye L. Correlation of adiponectin gene SNP +45T/G polymorphism and premature coronary heart disease of Han ethnic males. Natl Med J China. 2011; 91: 3413-6.

99. Sabouri S, Ghayour-Mobarhan M, Moohebati M, Hassani M, Kassaeian J, Tatari F, Mahmoodi-kordi F, Esmaeili HA, Tavallaie S, Paydar R, Sahebkar A, Tehrani SO, Ferns G, Behravan J. Association between 45T/G polymorphism of adiponectin gene and coronary artery disease in an Iranian population. ScientificWorldJournal. 2011; 11: 93-101.

100. Boumaiza I, Omezzine A, Rejeb J, Rebhi L, Ben Rejeb N, Nabli N, Ben Abdelaziz A, Boughzala E, Bouslama A. Single-nucleotide polymorphisms at the adiponectin locus and risk of coronary artery disease in Tunisian coronaries. J Cardiovasc Med (Hagerstown). 2011; 12: 619-24.

101. Chen G, Qin Q, Li Y, Ren B, Lu Y, Kou L, Yang N, Feng J, Zhao B. Association between plasma adiponectin and small, dense low density lipoprotein in patients with coronary artery disease. Chinese Journal of Cardiology. 2012; 40: 752-6.

102. Gui MH, Li X, Jiang SF, Gao J, Lu DR, Gao X. Association of the adiponectin gene rs1501299 G>T variant, serum adiponectin levels, and the risk of coronary artery disease in a Chinese population. Diabetes Res Clin Pract. 2012; 97: 499-504.

103. Shi K, Zhu Y, Miu Y, Guo X. Two single nucleotide poIymorphisms (+45T>G and+276G>T) of adiponectin gene and coronary artery diseases in the elderly without diabetes. Chin J Mult Organ Dis Elderly. 2012; 11: 187-91.

104. Zhang H, Wang D, Jin M, He X, Zhang Y. Serum adiponectin level and the diversity of gene SNP 276 in atherosclerosis. Chinese J of Health Lab Tech. 2012; 22: 2550-3.

105. Nan N, Jin Z, Yang Z. Genetic association of ADIPOQ gene polymorphisms with type 2 diabetes mellitus with coronary artery disease. J Capital Med Univ. 2012; 33: 6-11.

106. Antonopoulos AS, Tousoulis D, Antoniades C, Miliou A, Hatzis G, Papageorgiou N, Demosthenous M, Tentolouris C, Stefanadis C. Genetic variability on adiponectin gene affects myocardial infarction risk: the role of endothelial dysfunction. Int J Cardiol. 2013; 168: 326-30.

107. Rizk NM, El-Menyar A, Marei I, Sameer M, Musad T, Younis D, Farag F, Basem N, Al-Ali K, Al Suwaidi J. Association of adiponectin gene polymorphism (+T45G) with acute coronary syndrome and circulating adiponectin levels. Angiology. 2013; 64: 257-65.

108. Wang C, Piao L, Li D. Correlation between adiponectin gene +276 polymorphism and Carotid Intima Media Thickness in patients with Type 2 Diabetes Mellitus in Yanbian District. Chinese J Arteriosclerosis. 2013; 21: 721-6.

109. Wu H, Chen F, Shen J, Liu Y, Yang R. Relationship between adiponectin gene +276 nucleotide G/T polymorphism with coronary heart disease in Hui and Han nationalities in Ningxia. J Clinical Cardiology (China). 2013; 29: 836-9.

110. Cheung CY, Hui EY, Cheung BM, Woo YC, Xu A, Fong CH, Ong KL, Yeung CY, Janus ED, Tse HF, Sham PC, Lam KS. Adiponectin gene variants and the risk of coronary heart disease: a 16-year longitudinal study. Eur J Endocrinol. 2014; 171: 107-15.

111. Li Y, Qin Q, Ren B, Zhao L, Mao Y, Zheng P. The association between serum adiponectin levels and gene polymorphism with coronary artery disease in patients with essential hypertension. Tianjin Medical Journal. 2014; 42: 903-7,8.

112. Alehagen U, Vorkapic E, Ljungberg L, Lanne T, Wagsater D. Gender difference in adiponectin associated with cardiovascular mortality. BMC Med Genet. 2015; 16: 37.

113. Torres T, Bettencourt N, Ferreira J, Carvalho C, Mendonca D, Vasconcelos C, Selores M, Silva B. Lack of association between leptin, leptin receptor, adiponectin gene polymorphisms and epicardial adipose tissue, abdominal visceral fat volume and atherosclerotic burden in psoriasis patients. Arch Physiol Biochem. 2015; 121: 103-8.

114. Zhang M, Peng Y, Lv S. Association of genetic variants in the adiponectin gene and premature coronary artery disease. J Cardio & Pulm Dis. 2015; 34: 429-35.

115. Liu Y, Lin S. Study on relation between adiponectin and its gene polymorphisms and Yao coronary heart disease of Guangxi District. J Mol Lab Med. 2015; 30: 56-9.

116. Suo S, Sui X, Li X, Yang Z, Wang F. Interaction pf Polymorphisms of UCP2-866 G/A and ADIPOQ +45 T/G on the Risk of Type 2 Diabetes Mellitus with Coronary Artery Disease. Progress in Modern Biomedicine. 2016; 16: 2258-63.

117. Li S, Lu N, Li Z, Jiao B, Wang H, Yang J, Yu T. Adiponectin Gene Polymorphism and Ischemic Stroke Subtypes in a Chinese Population. J Stroke Cerebrovasc Dis. 2017; 26: 944-51.
